# Exploring the Materials and Condition of 20th-Century Dolls in Zoe Leonard’s *Mouth Open, Teeth Showing* 2000

**DOI:** 10.3390/polym15010034

**Published:** 2022-12-22

**Authors:** Judith Lee, Libby Ireland, Joyce H. Townsend, Bronwyn Ormsby, Angelica Bartoletti, Deborah Cane, Simoní Da Ros, Rose King, Isabella del Gaudio, Katherine Curran

**Affiliations:** 1 Conservation Department, Tate Britain, Millbank, London SW1P 4RG, UK; 2 UCL Institute for Sustainable Heritage, University College London, 14 Upper Woburn Place, London WC1H 0NN, UK

**Keywords:** twentieth-century dolls, plastic, degradation, conservation, contemporary art

## Abstract

Systematic condition and analytical surveys were carried out on Zoe Leonard’s (b. 1961) *Mouth Open, Teeth Showing* 2000, an installation artwork in Tate’s collection consisting of 162 children’s dolls. The dolls were manufactured at various points within the 20th century and encompass several potentially problematic synthetic polymers found in modern and contemporary museum collections. To explore the doll materials and conservation condition, a multi-analytical approach was used to identify key synthetic polymer types and additives present, including portable and bench analytical techniques. Challenging degradation phenomena associated with different types of doll have been discussed and related to their material composition, which has helped our understanding of the conservation challenges inherent to this contemporary artwork.

## 1. Introduction

Zoe Leonard, b. 1961 is an American artist who works with photography and sculpture where found or abandoned objects appear frequently in her works. *Mouth Open, Teeth Showing* 2000, is an installation artwork consisting of 162 children’s dolls collected by the artist at flea markets in 1999. The dolls are displayed as the only artwork in a gallery space, with the dolls standing upright, arranged in a grid pattern and all facing the same direction according to the artist’s specification (images of the artwork on display are available online [1]), see also Supplementary Information File 1 (S.I.), Figure S1). Leonard became interested in the dolls because of the visible impact of age and past use, i.e., evidence of play and associated modifications, on their appearance which imparts a degree of individuality onto otherwise mass-produced objects, whilst the title of the work mixes vulnerability and menace.

The dolls also present a record of changing fashions, gender roles, and diversity of representation over time. This in turn speaks to the feminist narrative with the group showing solidarity and the capacity for action as one [1]. The artist has done little to restore or repair wear to the dolls, making interventions only to ensure they can stand up: some dolls have adhesive placed in joints to stiffen them; some feet originally shaped to fit high-heeled shoes have been modified to create a flat sole; and soft-bodied dolls have had wire armatures placed internally to hold them upright. In past installations the feet of each doll were hot-glued to the floor so the dolls appeared free-standing, as though they had independently gathered in the space.

Precise dating and identification (i.e., identifying the manufacturer and model) of the dolls in *Mouth Open, Teeth Showing* was challenging as the dolls typically lack original packaging, clothing or makers’ marks. However, dates of manufacture may be approximated based on materials analysis and stylistic observations with reference to books and websites written by, and for, doll collectors and enthusiasts [2,3,4,5,6,7].

In accordance with established art acquisition procedures at Tate, a condition survey of the dolls was carried out to inform decision making for acquisition into the collection along with ongoing handling, display and storage requirements for this artwork. The objectives of the combined condition and analytical surveys were to document the condition of all 162 dolls at the point of acquisition and to identify dolls that appeared most vulnerable to further degradation (which may then require augmented conservation and storage approaches). Another key objective was to identify the polymeric materials and applied paint layers present across a representative sub-set of 44 dolls using both portable and micro-destructive scientific analytical techniques (Section 2.2), with a view to facilitating ongoing display of the artwork.

In the case of *Mouth Open Teeth Showing*, preliminary discussions held with the artist upon acquisition of the artwork established that Leonard is aware that the dolls will continue to deteriorate over time, and that she considers evidence of age and change intrinsic to the artwork. Leonard is also aware that the light levels used for past displays of the artwork could impact on rates of deterioration, which suggests some acceptance of possible colour change and fading. However, at present it is not known how familiar the artist might be with the unavoidable and ongoing changes associated with different dolls made from varying polymeric materials. Nor is it known to what extent she is accepting of further change, and whether she is concerned about changes that could disproportionately affect the appearance of specific elements of individual dolls. Furthermore, in the long term it is anticipated that individual dolls will require replacement, and discussion with the artist will be fundamental to identify appropriate end points, and sympathetic approaches to conservation. In addition to working toward a better understanding of the artist’s intention and how to balance this with the preservation and conservation needs of the artwork, the condition and analytical survey also offered a unique opportunity to contribute to the technical scholarship of 20th-century dolls. Prior to this study, there were no combined condition and analytical surveys which utilised the suite of analytical techniques employed, no studies involving a significant number of 20th-century dolls produced by various manufacturers, and none which considered the findings within a contemporary art context, and in particular where the artwork involved the re-use of old dolls.

### Materials Used in Doll Manufacture

Children’s toys and dolls are manufactured using natural and synthetic materials developed primarily for industrial and technological applications. Since the chronological development of synthetic polymers is well documented [8,9,10,11] the identification of the materials used to make a particular doll can help inform likely dates of manufacture or approximate age. Dolls belonging to the late 19th century were often made using natural/non-synthetic materials, e.g., ‘one-piece stuffed-body’ constructed dolls with heads made from bisque (a ceramic material fired at a relatively low temperature) or porcelain [2]. The use of dyed mohair, human hair and boar bristles for making dolls hair and eye-lashes has been documented [10]. From 1892, composition dolls were made using wood-pulp which created a more robust doll relative to those made from *papier-mâché* [2], and composition dolls became very popular in the 1920s. The semi-synthetic polymers cellulose nitrate (CN, available from 1870) and cellulose acetate (CA, available from 1905) were also both used to make dolls; between 1900 and the mid-1930s, CN (made from Celluloid™) dolls were common [8]. From the 1940s, there was an interest in producing ‘drink-and-wet’ dolls [2] that could tolerate water exposure, which combined with manufacturing developments, led to the widespread use of plastics such as polyvinyl chloride (PVC, available from 1927), polystyrene (PS, available from 1937), low density polyethylene (LDPE available from 1941) and high-density polyethylene (HDPE, available from 1957) [8]. From the 1950s, vinyl heads with rooted saran (vinyl chloride/vinylidene chloride copolymer, VC/VDC) hair were common [2,12,13].

The manufacturing of plastic dolls was often achieved by injection moulding, typically with the doll made in two halves, which were then glued together [2]. Blow-moulded plastic dolls were also made, which were not as thick or hard as injection-moulded dolls and did not require gluing. Soft and flexible vinyl dolls could be produced by rotational moulding [2]. The materials used for clothing and accessories also varied, however cotton, leather, wool and nylon were all commonly used in the 20th century [10] and indeed any common synthetic fabric, e.g., Nylon or acrylic, used in the clothing industry could be used.

Even though children’s dolls and toys can form significant parts of museum collections (as examples, 2451 objects listed as Dolls & Toys on the V&A website https://collections.vam.ac.uk/search/?q=Dolls&page=1&page_size=15&id_category=THES48967 (accessed on 19 January 2021) and there are many museums and associations specialising in antique dolls and toys, etc.—see ‘Caring for Dolls & Toys, Smithsonian Museum Conservation Institute’, https://www.si.edu/mci/english/learn_more/taking_care/dolls.html (accessed on 19 January 2021), there are relatively few studies published in the conservation literature which document degradation phenomena and treatment options for 20th-century dolls [12,13,14]. For CA dolls, issues with ‘sleep-eyes’ (weighted eyes that close when the doll is placed lying down horizontally) have been documented where any iron-containing mechanisms become stiff, preventing the dolls eyes from opening when held vertically. Brown accretions may also be visible around the eyes. Iron is known to catalyse the degradation of CA and reacts with acetic acid to form iron acetate [15] which can contribute to the occurrence of sleep eyes, becoming stiff [15]. In some cases, dolls with sleep eyes have been inappropriately stored on their backs, causing the counterweight of the eye mechanism to become stuck in the interior of the head, preventing the eyes from opening at all [14]. Other reported degradation phenomena include the formation of white stearic acid bloom on PVC components [8] and stearyl alcohol bloom in PVC dolls from the 1980s and 1990s [16]. Surface whitening and migrated plasticiser on PVC elements of 20th-century Barbie dolls have also been reported [13]. Thus, in general, the identification of the main polymer types present in each doll remains key to informing conservation strategies which often involve minimising exposure to ultraviolet (UV) light, preventing exposure to fluctuating environmental conditions and utilising cool storage as appropriate [9].

## 2. Materials and Methods

The condition and analytical surveys were all carried out within a limited period in early 2019, when the artwork was available for study at Tate’s storage facility, and where visual examination, in situ non-destructive analysis and targeted micro-sampling was also possible.

### 2.1. Appearance and Condition Surveys

Two condition surveys were carried out in sequence. The first was a broad condition survey (Section 2.1.1) which involved visual examination of all 162 dolls, including documentation of key observations. Based on this broad survey, 44 dolls were selected for closer examination of condition, where a greater number of visible parameters and other observations (e.g., smell) were systematically recorded (Section 2.1.2), and materials were characterised (Section 2.2). The methodologies employed for the surveys, and recorded parameters, are described below.

#### 2.1.1. Broad Condition Survey of All Dolls

A broad survey of all 162 dolls was carried out by conservators at Tate’s storage facility using the naked eye, with and without the aid of Optivisors, and with illumination provided by portable studio lighting (tungsten-halogen lamps). Incidences of the following key parameters were systematically recorded for each doll: surface dirt, scratches, abrasions, cracking, embrittlement, discolouration (e.g., possible yellowing), surface bloom, weeping, sweating, sticky exudates, and the presence of strong or distinctive smells. Other observations which may provide clues about manufacturing methods and/or materials were also documented, including manufacturers’ marks, striations on the surface, moulding lines, a rubbery or plasticised feel, or whether the material felt rigid. This survey helped to identify the dolls that were in a more advanced state of degradation and in need of a tailored conservation strategy and informed the identification of a sub-set of 44 dolls for detailed materials analysis (Section 3.3). The 44 dolls were chosen to be representative of the artwork as a whole, i.e., the selection included a range of likely ages, possible polymer types, plastic colours, conditions, visible degradation phenomena, and installation location when on display (see S.I., Figure S1).

#### 2.1.2. Detailed Condition Survey of Selected Dolls

For the 44 selected dolls a further, more detailed condition survey was performed. Surveys drew on the approach described for synthetic polymeric objects by Keneghan [17] and on past surveys designed by the Birmingham Museum Trust (BMT) in collaboration with the National Trust. This survey was designed to capture an extensive and specific series of observations pertaining to degradation and condition, and to include an overall condition metric to categorise each doll. Observations relating to condition were recorded for the selected dolls (Section 3.1), documenting observable damage or evidence of ageing such as discoloration, embrittlement, blooming, sweating, crazing, scratches/abrasions. The dolls were assigned overall condition ratings which ranged from 1 (Excellent/Stable) to 4 (Poor/Highly unstable), as defined in Table 1 below. Condition scores were provided for both the artefact as a whole and for individual plastic components within a single doll.

### 2.2. Analytical Methods

Material analysis included non-destructive near-infrared reflectance spectroscopy (NIR) and external reflectance-Fourier transform infrared (ER-FTIR) spectroscopy, with targeted sampling for transmission FTIR, energy dispersive X-ray spectroscopy (EDX), cross-sections observed at ×100–320 magnification in visible and UV light, fibre identification for clothing or hair, and pyrolysis gas chromatography mass spectrometry (PyGCMS). These analytical techniques were used primarily to identify the key polymer types, however in many cases analysis they also permitted the identification of pigments, extenders and additives. FTIR, EDX and PyGCMS were generally utilised to characterise surface exudates or efflorescence, or localised areas and surfaces of interest that would not necessarily be characterised using NIR and ER-FTIR techniques [18].

NIR was carried out using an ASD LabSpec 5000 spectrometer (Boulder, CO, USA), equipped with a 2 mm spot diameter fibre optic cable, and measuring in the 350–2500 nm range. The spectral resolution of the instrument was 3 nm at 700 nm and 10 nm at 1400–2100 nm, while the sampling interval was 1.377 nm at 350–1050 nm and 2 nm at 1000–2500 nm, with a scanning time of 100 ms. The spectra were analysed using the method described by Zhu et al. [19]. Spectra in the range between 900 and 1700 nm were normalized, derivatised (using the 1st derivative), and smoothed, before proceeding with Principal Component Analysis (PCA) using OriginPro 2020b software (version 9.9, Academic, OriginLab Corporation, Northampton, MA, USA) using the Factor Analysis (v1.00) plug-in, by reducing and extracting the spectra components as a covariance matrix. PCA was applied to reduce the dimensions of the spectral dataset, enabling the cumulative contribution rates of the first 25 principal components to explain the variance.

ER-FTIR spectra were collected on a Bruker Alpha II spectrometer equipped with the External Reflectance QuickSnap module using OPUS v7.5 software (Bruker Optik GmbH, Ettlingen, Germany). All spectra were recorded in the range of 4000–399 cm^−1^, with a spectral resolution of 4 cm^−1^ and 16 co-added scans. Spectra were compared to an in-house library (see S.I., File 2) using OPUS v7.5 software, and the PolIReS total reflectance library [20,21,22] using Spectragryph v1.2.16 software [23]. In some cases, the Kramers-Kronig (KK) transformation was applied to the spectra to better distinguish between similar polymer types such as polystyrene (PS) and acrylonitrile butadiene styrene (ABS) and to corroborate the NIR identification.

Transmission FTIR was carried out using a Thermo Scientific Nicolet iN10 MX microscope (Thermo Scientific™, Altrincham, UK) equipped with a liquid nitrogen cooled mercury-cadmium-telluride (MCT)-A detector (Thermo Scientific™, Altrincham, UK). Samples removed from the dolls were applied to a single diamond cell and rolled flat using a steel roller. 64 scans were collected at a resolution of 4 cm^−1^ across a wavenumber range of 4000 to 600 cm^−1^. Data was processed using Omnic 9 software. Several separate areas were analysed per sample and representative spectra were chosen for interpretation.

EDX of samples removed from the dolls was carried out using a JEOL IT-500 SEM (JEOL, Tokyo, Japan) and Oxford INCA software (Oxford Instruments PLC, Abingdon, UK), using 20 kV, a working distance of 10 mm, 30 Pa air and a back-scattered electron detector. Samples for EDX were analysed without any coating.

PyGCMS was carried out using a multi-shot pyrolyser EGA/PY 3030D (Frontier Lab, city, country) coupled with an Agilent 8890 gas chromatographic system (Frontier Laboratories Ltd., Fukushima, Japan) with a split/splitless injection port and an Agilent 5977B mass selective single quadrupole mass spectrometer. Samples were placed into deactivated pyrolysis sample cups (PY1-EC80F, Eco-Cup LF). For derivatisation of selected samples only, 5mL of hexamethyldisilazane (HMDS) from Sigma-Aldrich (Merck Life Science UK Limited, UK) was added to the sample in the sample cups. The interface to the GC was held at 320 °C and purged with helium for 30 s before opening the valve to the GC column. The samples were dropped into the furnace and pyrolyzed at 550 °C for 0.5 min. The pyrolysis products were transferred directly to an Ultra ALLOY-5(MS/HT) (Frontier Laboratories Ltd., Fukushima, Japan) capillary column (30 m × 0.25 mm × 0.1 mm) with the helium carrier gas set to a constant flow of 1.0 mL/min. Injection with a 30:1 split was used. The GC oven temperature program was 40 °C for 1 min; 10 °C/min to 320 °C; isothermal for 1 min. The Agilent 5977B MSD (Agilent Technologies, California, USA) conditions were set as follows: EI, 70 eV, transfer line at 320 °C, MS Quad 150 °C, MS Source 230 °C, electron multiplier at approximately 954 V; scan range 33–550 amu.

## 3. Results and Discussion

Information gleaned from the condition survey of all 162 dolls is described below in Section 3.1. For the 44 dolls selected for closer study, Section 3.3 provides an overview of the main polymer types identified (Table 2, Table 3, Table 4, Table 5 and Table 6). The selected dolls were grouped into categories based on the main/distinguishing polymer types identified. These categories of doll are described and discussed in detail in Section 3.4, which also incorporate observations from the detailed condition survey. A specific discussion has been devoted to PVC in Section 3.4.4, since this material was used across many of the assigned categories of doll and often showed clearly visible ageing phenomena. Although not the main target of the study, some attention has also been given to clothing and hair in Section 3.4.6. Finally, implications of these results for ongoing storage and display have been explored in Section 3.5.

### 3.1. Appearance and Condition Surveys: General Findings

**Figure 1:**
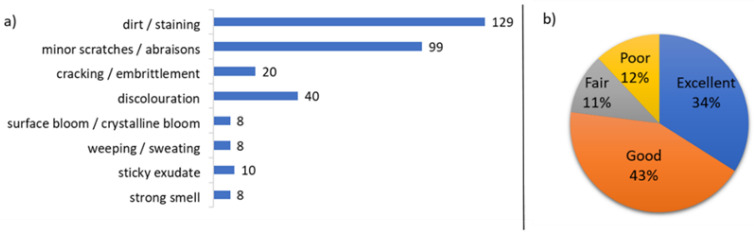
(**a**) Distribution of key observations from the condition survey of all 162 dolls in Zoe Leonard’s *Mouth Open, Teeth Showing* 2000. The numbers of dolls presenting the specified observations were recorded to gain an insight into common and less common ageing and degradation phenomena. (**b**) Summary of the proportion of all 162 dolls classed as being in excellent, good, fair and poor condition according to the condition metric defined in Table 1. All observations were recorded in 2019.

A summary of key observations and overall condition of all 162 dolls is presented in Figure 1 below. Specific observations according to polymer type have been discussed within the analytical summaries below (Section 3.4). As seen in Figure 1, using the metric defined in Table 1 (above), the majority of dolls were classified as being in excellent or good condition upon acquisition, whilst ~25% of the dolls were in fair or poor condition. Dolls in better condition tended to have only visible surface dirt, minor scratches and abrasions documented in the condition survey, often with matted hair that appeared otherwise in good condition.

#### 3.1.1. Discolouration

Forty dolls, or around 25% of the total, had components that appeared discoloured to the naked eye (Figure 1a). Suspected discolouration was noted where flesh tones of the plastic components had an unnatural yellow or orange appearance, where limbs appeared a very different colour from the torso but appeared to be a similar material, and in cases where areas that may have experienced more light exposure (a known agent of deterioration for plastics) were a different colour to equivalent covered areas. It is noted however that any colourants present in the plastic components were typically not identified using the suite of analytical techniques employed.

The polymeric components associated with possible discolouration were characterised in thirteen of the forty discoloured dolls. Of these, two composition dolls had discoloured nitrocellulose paint layers and the discoloured plastics included PE (in two dolls, Table 3), PVC (in five dolls, Table 5), and PS (in four dolls, Table 6). For those dolls with discoloured components that were not analysed it was possible to assign a likely polymer identification based on observation of surface properties, and comparison to the dolls that were analysed. Twenty-two dolls featured discoloured heads (likely to be PVC in many cases); 17 dolls had discoloured arms (likely to be PVC in many cases), and 14 dolls had discoloured legs (likely to be PE in many cases).

#### 3.1.2. Physical Weakness

Twenty dolls showed evidence of cracking or embrittlement (Figure 1a). Cracked or embrittled components were identified in four composition dolls, two CA dolls (Table 2), one doll made with PE (Table 3), and two PS dolls (Table 6). Of the plastic dolls with cracked/embrittled components, the areas most affected were torso and legs made of rigid plastics. The composition dolls that were embrittled showed cracking throughout the bulk moulded material making up the structure of the dolls, as well as in applied paint layers, indicating that the composition dolls can become extremely vulnerable over time.

#### 3.1.3. Smell

Eight dolls were noted as having a distinct smell whilst unpacking. In some cases, a vinegar smell was apparent which is often considered indicative of CA degradation [24], and indeed three of these dolls were analysed and confirmed to contain CA components (Section 3.4.2). In other cases, a smell interpreted as being associated with plasticisers and reminiscent of inflatable PVC objects was noted, along with an acidic smell, possibly associated with PVC [25,26]. Given the subjective nature of smell, there was difficulty recording distinct yet unfamiliar smells, particularly those that dissipated during examination. It was not always possible to identify the component of a given doll from which the smells were originating. It is interesting to consider that the smell of the dolls could be considered an intangible but important part of the artwork [25,27,28]. Analysis of volatile organic compounds (VOCs) [27,29] was out of the scope of this study, however this has been identified as a potential avenue for further investigation, particularly for the more pungent dolls as it could provide further insight into composition and degradation pathways and states.

#### 3.1.4. Surface Exudates

For a small number of dolls, surface exudates were visible, with eight dolls presenting white surface bloom or crystalline deposits. A further eight dolls presented ‘sweating’ surfaces, and ten dolls were tacky to the touch. Some dolls were both sweaty and tacky to the touch. Analysis of the white surface crystals and sticky exudates primarily identified migrated plasticisers (Section 3.4.4). White crystalline surface deposits were noted on the head of one doll were characterised as PVC (Table 5), and a white surface bloom was most noticeable on three dark-coloured dolls’ heads, suspected to be made of PVC, although not analysed in this case. Two CA dolls had crystalline deposits, and one CA doll with a head made of PVC) had weeping/stickiness visible across the face (Table 2). One doll with a PS body and another with PE legs were also noted as having sticky surfaces on the PS and PE components, possibly indicating migration of plasticisers (Table 3).

Monitoring the dolls that present surface bloom, and exudates indicative of plasticiser loss, will be important since loss of plasticiser can lead to mechanical vulnerability and deformation [30]. In addition, tacky surfaces will be more prone to dirt pick-up when the dolls are on display, while continued migration of plasticisers or other additives, could eventually become distracting. Judging the point at which an intervention such as surface cleaning may become desirable would ideally be partly informed through engagement with the artist and balancing risk vs. aesthetic gains.

#### 3.1.5. Mounting

A significant source of damage to ~70% of the dolls related to the past use of hot glue to secure the dolls’ feet to the gallery floor as the mounting/display method; this was the display method directed by the artist to help create the illusion of movement and striding forth of the army of dolls [1]. 113 of the 162 dolls had damaged feet, with areas of feet that appeared to have softened during application of the hot glue, and with breaks or losses presumably caused by subsequent mechanical detachment from the floor during de-installation. 137 dolls had colourless adhesive residues visible on the feet (see Section 3.5.1). It was noted that 47 dolls would be unable to stand upright safely if they were secured only at the base of their feet. Further work (Section 3.5) is required to devise a suitable and discreet mounting method(s) for the dolls which minimises potential additional damage.

### 3.2. Dating and Makers’ Marks

It was possible to date 29 of the 44 dolls selected for analysis based on makers’ marks that were visible on the backs of heads (e.g., S.I. Figure S2) which included information such as the company name, year of manufacture or a patent number. Several manufacturers were identified across the 44 dolls (S.I., Table S1): The Reliable Toy Co. est. 1920 in Toronto, Canada in [2]; the Ideal Novelty and Toy Co., Queens, USA est. 1907 [31]; Uneeda Dolls, New York, USA est. in 1917 [32]; Remco Inc. New Jersey, USA est. in 1949 [33]; GéGé dolls, Moingt, France est. 1934 in [34]; Migliorati, Italy; Furga, Italy, est. 1872 [35]. The artwork includes dolls manufactured in Canada, USA, China, Italy and France (S.I., Table S1). For dolls without makers’ marks, estimated dates of manufacture were assigned based on identified polymer types, comparison with dolls of known dates, and apparent fashion or style. The majority of the dolls in the artwork are believed to date from the 1950s to 1970s, although there is a smaller number of early (pre-1950s) and later dolls (~1980s–90s).

### 3.3. Summary of Key Polymer Types and Additives Identified in the 44 Selected Dolls

For the 44 selected dolls, the polymer types comprising key structural elements of the dolls (heads and/or bodies) and some hair/clothing were identified (see S.I., Table S1). Polymers used for heads and/or bodies included PE, PS, CA, polypropylene (PP), and polyvinyl chloride (PVC). Other materials identified included CN used for paint layers. Hair fibres were made using polyamide (PA), PP and wool. It was common for a single doll to contain at least two different plastic types. PyGCMS was also carried out on selected samples (see S.I., Table S2) which helped to identify some common phthalate plasticisers and other additives, as discussed below.

### 3.4. Key Findings Per Doll Type

#### 3.4.1. Composition Dolls

**Figure 2:**
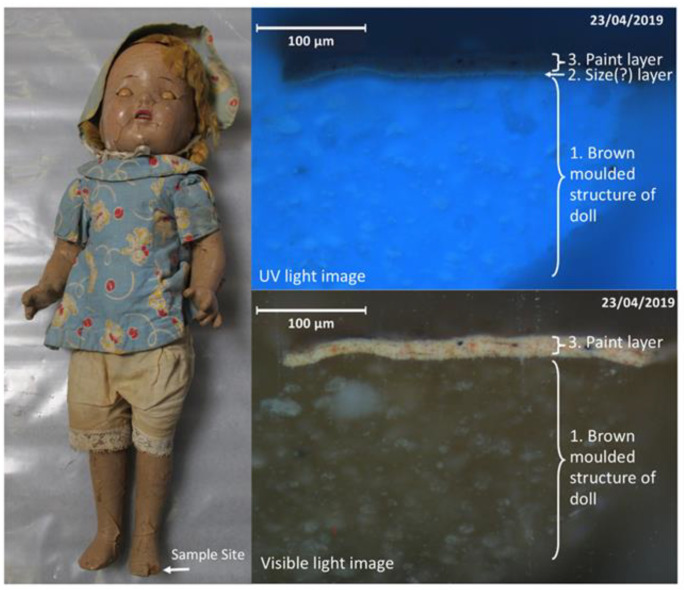
An example of one of the composition dolls (I10) with a severely cracked and delaminating nitrocellulose paint layer. Cross sections (shown in UV light and visible light) taken from a loss in the doll’s foot show three main layers: 1. the main structure of the doll comprised of brown moulded material 2. A green-white UV-fluorescent layer that could indicate a glue size layer (only visible in the UV cross section image) and 3. a single paint layer ~25 um thick. Image ©Tate.

Four of the selected dolls were made using ‘composition’ (Table S1, S.I.), an example shown in Figure 2 below. Composition is a brown fibrous material that is moulded into the desired shape and has a similar appearance to cork. Composition dolls were manufactured from ~1909–1950s [36]. FTIR analysis of samples taken from the brown moulded structure (accessible in areas where there are breaks and losses) of doll I10 identified cellulosic material and a natural resin (for spectra see S.I., Figure S3 and Table S3). Further analysis would be required to fully characterise the organic composition of the moulded material, however it is broadly consistent with reported composition mixtures. Recipes used for producing composition moulding material varied between doll-makers, but generally contained mixtures of sawdust, glue, and other materials such as corn starch, resin and wood flour, which would be shaped using a hot or cold mould [36]. This mixture is distinct from ‘compo’ used to make nineteenth-century decorative frames, which is typically a mixture of chalk, resins, glue (e.g., animal/hide glue, pearl glue) and linseed oil [37], although French compo may contain some paper pulp [38]. The analysis of the four composition dolls implies that a further two unsampled dolls in the larger group are likely to be composition dolls as they have similar characteristics, with cracking and lifting paint revealing an underlying brown fibrous material.

Composition dolls would typically be painted flesh colour [36]. Cross-sections taken from an area of damage on the left foot of composition doll I10 showed that the bulk moulding material had a bright blue UV fluorescence (Figure 2, layer 1, UV light image) and a very thin and UV fluorescent layer that appeared greener (Figure 2, layer 2, UV light image) was situated just beneath the paint layer which could indicate the presence of a glue size layer. The pink paint layer (Figure 2, layer 3) was ~25 µm thick. FTIR analysis of the paint layer identified nitrocellulose (see S.I., Figure S4 and Table S3) with the presence of camphor indicated by a carbonyl band at 1722 cm^−1^ [39], which was used as a plasticiser and flame retardant for early nitrocellulose formulations [40]. EDX analysis (see S.I., insert Figure S4) of the paint layer identified a substantial proportion of titanium (Ti), consistent with titanium white pigment. The presence of iron along with traces of magnesium, aluminium and silica is indicative of an earth pigment, with calcium and phosphorus indicating the presence of bone black. EDX analysis also indicated traces of lead, zinc and chromium, indicating the possible presence of zinc oxide, lead chromate and/or lead driers, or lead white in the paint.

All composition dolls examined in the broad condition survey presented fragile surfaces, with widespread cracking of the paints. Doll I10 (Figure 2) showed significant lifting and losses of the paint layer, particularly around the feet, which could in part relate to damage caused by previous mounting methods. NC paint formulation aspects such as solids content, plasticiser and/or flame-retardant content (e.g., the use of phthalates, tricresyl phosphate, and triphenyl phosphate) and the presence of other resins (e.g., alkyd, ketone, urea, maleate and acrylic) can all impact film flexibility and crack resistance [41]. Other important factors include the unknown historical environmental conditions (UV and visible light, temperature, relative humidity) encountered by each doll in addition to age and degree of handling by past owners, as well as repeated installation and deinstallation, which together have contributed to the differing states of preservation.

The composition dolls did not feature any makers’ marks, hence assigning approximate dates of these dolls relies upon materials analysis. Nitrocellulose paints were developed from 1918, and were widely available by the mid-1920s, commonly used as car or household paints [42,43]. The use of nitrocellulose paint on the composition dolls, and the use of titanium white pigment indicates an earliest date of manufacture as the 1920s. It can be presumed that for the reasonably large-scale production of composition dolls, the quick drying nature of nitrocellulose lacquers and ability to utilise spray or dipping paint application [41] and rapid painting [42] would have been advantageous.

#### 3.4.2. Cellulose Acetate (CA) Dolls

**Figure 3:**
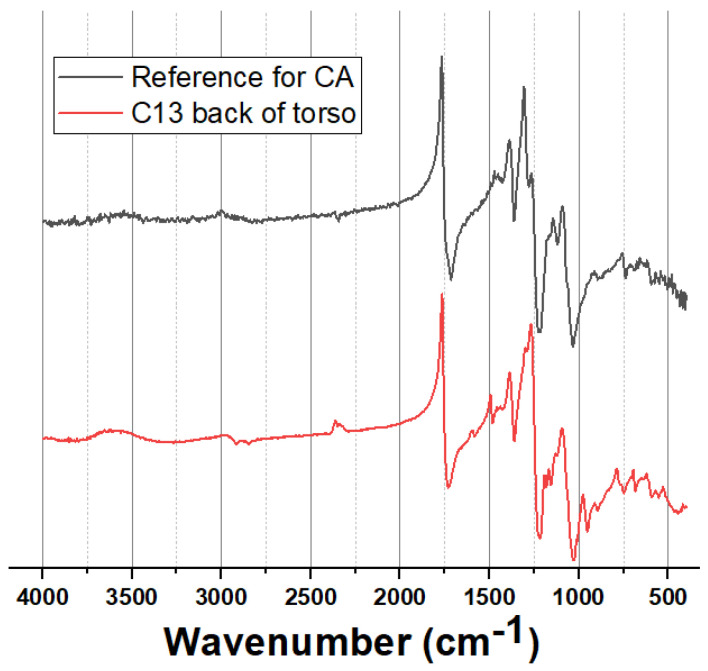
Typical ER-FTIR spectrum for CA dolls, illustrating the obtained spectrum for doll C13 (back of torso) and a reference spectrum for CA.

Five of the 44 dolls had torsos and limbs made of CA, sometimes made with heads made of PVC (S.I., Table S1). A typical CA ER-FTIR spectrum obtained is shown in Figure 3.

CA was initially developed in 1869 as a less flammable alternative to CN which had been used to make children’s dolls between 1900–1930 [8]. Four of the CA dolls had dates between the 1950 and 1970s (Table S1, S.I.), but are certainly no earlier than 1908, when injection moulding for CA was developed for making various products including toys [44]. CA dolls with PVC components will date from after 1927, when plasticised PVC was introduced [8]. Plastics-grade and fibre-grade CA are reported to have a slightly lower degree of substitution (2.5–2.7) relative to CA used as a film substrate (2.9–2.92) [45]. CA degrades through a combination of processes, including hydrolysis which results in deacetylation, cleavage of the glycosidic bonds, plasticiser loss, oxidation and photodegradation [8,46,47].

A summary of the condition of the 5 dolls found to have CA components is given in Table 2. These showed varying stages of deterioration, with those showing more advanced stages of deterioration not necessarily being the oldest dolls. Two CA dolls exhibited cracks or breaks and losses, possibly the result of impact damage, reflecting the relatively brittle nature of CA. Areas of the CA dolls that appeared more degraded tended to have crazed matte surfaces, sometimes slightly grey in colour and with a powdery surface. Better preserved areas of CA dolls had smoother glossier surfaces without crazing, and also appeared less bleached relative to the more degraded areas. The CA dolls tended to have a vinegar smell indicative of deacetylation and release of acetic acid [45]. Comparison of FTIR spectra of well-preserved vs. visibly degraded areas of CA doll C13 (S.I., Figure S5a vs. S5b, Table S4) showed that degraded CA had a far more prominent OH band centred at ~3400 cm^−1^, diminished bands at 1719, 1237 and 1372 cm^−1^, which are all consistent with loss of acetyl groups [45].

**Figure 4:**
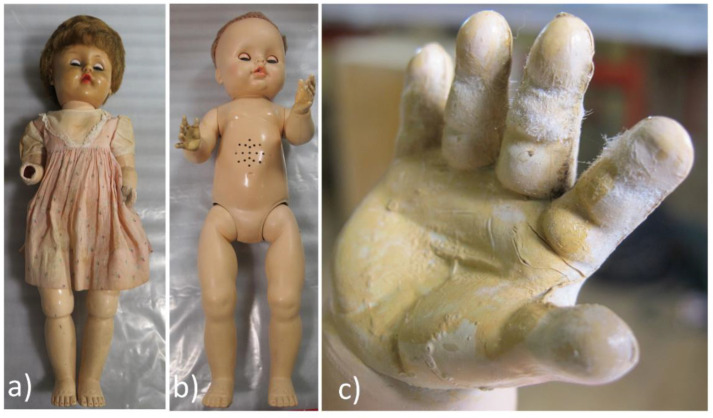
Degradation of CA dolls: (**a**) doll K2 with a yellowed and degraded PVC head; (**b**) doll C13; (**c**) detail of the hand of doll C13 with needle-shaped crystalline efflorescence. Image ©Tate.

PyGCMS of a sample of plastic taken from the degraded hip joint plastic of CA doll C13 identified diethyl phthalate (DEP) and triphenyl phosphate (TPP) (see S.I. Figure S6 and Table S5), both of which are commonly encountered external plasticisers in CA artefacts [48], with TPP also having flame retardant properties [26,27]. CA doll C13 also had distinct needle-shaped white crystalline efflorescence visible on the hands (see Figure 4 below). EDX analysis found the crystals to be rich in carbon, phosphorus and oxygen (data not shown). Transmission FTIR of the crystals suggested a good match for TPP (S.I., Figure S5c and Table S4). This was confirmed by PyGCMS where TPP was identified, but DEP was not found as a component of the crystalline efflorescence, despite DEP being a component in the bulk plastic of the doll (see S.I., Table S2: C13, S3). This may be expected since DEP is volatile whilst TPP is solid at room temperature [49]. Similar crystalline efflorescence consisting of TPP has been identified on 20th-century CA toy soldiers [50] and CA film [51] and in CA industrial products including Rhodoid™, which were used by constructivist sculptors such as Naum Gabo, László Moholy-Nagy and Antoine Pevsner [52,53].

#### 3.4.3. Polyethylene Dolls with PVC Components

**Figure 5:**
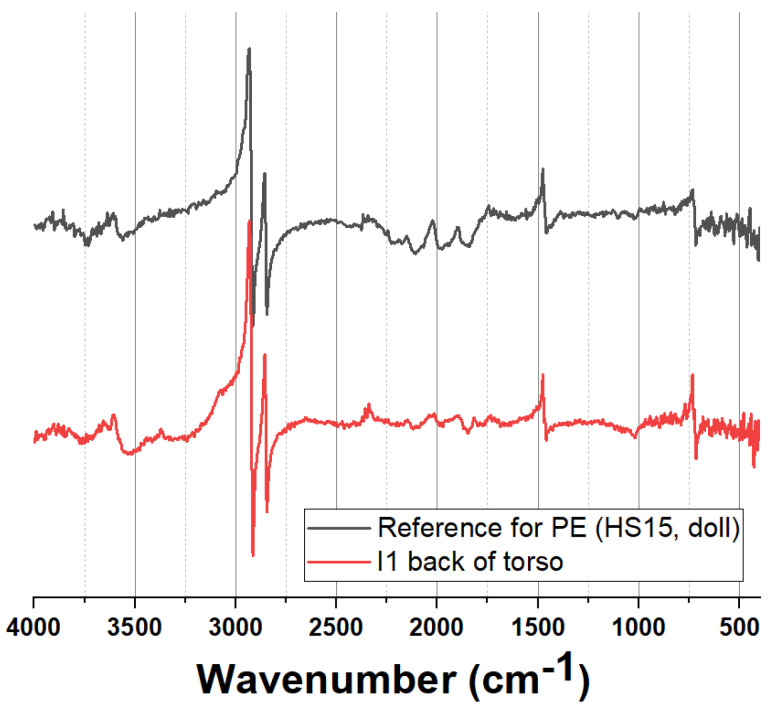
Typical ER-FTIR spectrum for polyethylene dolls, illustrating the acquired spectrum for doll E1 (back of torso) and a reference spectrum for PE.

The bulk of the dolls analysed were dated 1950s–1970s and had torso and legs made of PE (characterised using ER-FTIR and NIR) with PVC heads and arms. PE was readily identified using ER-FTIR and a typical ER-FTIR spectrum is shown in Figure 5.

**Figure 6:**
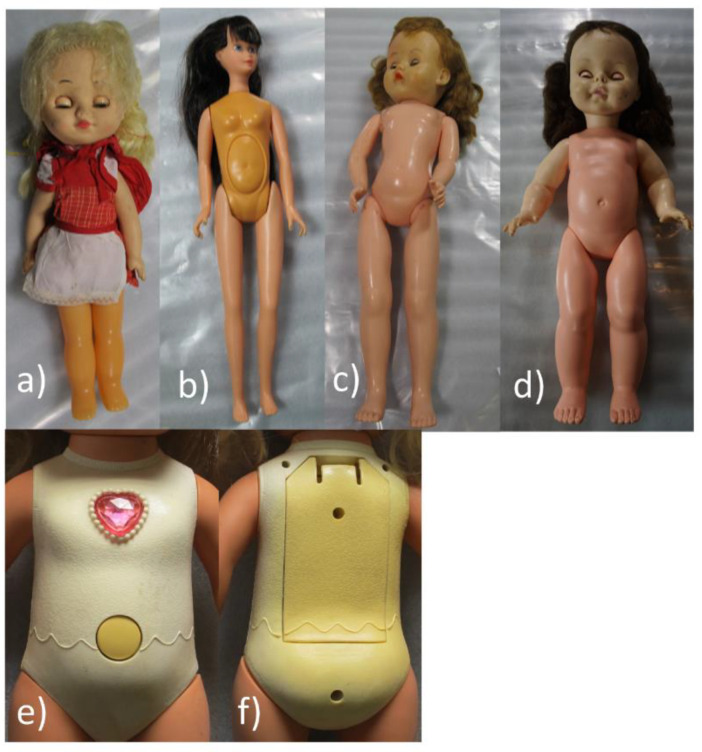
Dolls showing discolouration. (**a**) Doll G8 constructed with PE torso, legs and PVC arms and head. The legs have become discoloured and yellowed with age. (**b**) Doll I8, featured a discoloured PS torso (**c**) doll E6 with CA body, arms and legs and a discoloured PVC head. (**d**) Doll H7 with PE legs and torso, discoloured PVC arms and legs (**e**) Doll B4 with PS front (less yellow) (**f**) Doll B4 with PS back (more yellowed). Image ©Tate.

As detailed in Table 3, PE dolls/components were generally in good condition, although minor scratches and abrasions were commonly seen, and there was some evidence of embrittlement with occasional breaks or cracks. Detached or missing limbs were also seen. Portable ER-IR and NIR were largely successful in identifying PE, precluding the need for physical sampling for polymer identification, although it can be challenging to distinguish between HDPE and LDPE using infrared spectroscopy [54]. HDPE is typically used for thicker and more rigid structures whilst LDPE is used for flexible thinner plastic bags etc, hence HDPE is expected for these dolls. Although PE is considered to be amongst the group of more inert plastics, PE degrades via thermo-oxidative and photo-oxidative pathways, resulting in the formation of vinyl and carbonyl groups, and cross-linking and chain scission can occur, the latter resulting in the reduction of polymer molecular weight and loss of strength [55,56]. Discolouration of PE was suspected in several dolls, exemplified by doll G8 (Figure 6a), where the plastic for the legs appeared a distinctly orange colour, although further exploration is required to investigate the possibility of orange colourants in this plastic.

#### 3.4.4. PVC Components

Due to the poor quality of the spectra (i.e., noise), often caused by the geometry of the object being measured, interpretation of ER-FTIR spectra could be challenging. In particular the identification of PVC using ER-FTIR was complicated due to noise in the region where C-Cl absorption bands are expected. The use of complimentary NIR spectroscopy with PCA analysis was helpful for characterizing polymeric materials and for identifying PVC where ER-FTIR analysis was not clear. Principal components (PC) 1, PC2 and PC3 accounted for 54.1%, 22% and 15% of total variance, respectively. The first three principal components were used to draw a three-dimensional scatter plot (see S.I., Figure S7) to perform cluster analysis of measurements carried out on the dolls, where reference spectra aided polymer identification. Further to this, combined transmission FTIR and EDX analysis of samples taken from the artwork provided additional evidence for PVC components (e.g., arms and heads, some accessories) where phthalate plasticizers and a high amount of chlorine were detected, respectively.

EDX analysis of 11 PVC samples taken from 11 dolls dating between the 1950s and the 1980s was carried out (Table 4). Trace amounts of calcium, sulphur and silica were typically identified suggesting the presence of chalk and/or gypsum and silica as extenders. However, chalk was identified using FTIR for only a small number of the PVC samples and kaolin in only one sample (Table 4). The possibility of small amounts of other calcium-containing additives could not be excluded, e.g., calcium stearate which has been used as a heat stabilizer for PVC [57].

A summary of the condition of dolls with PVC components can be seen in Table 5. Frequent issues with PVC components included dirt adhesion, discoloration, and sticky exudates associated with plasticiser migration.

The plasticiser content of PVC can comprise a considerable proportion of the overall plastic composition, up to ~50% by weight [58,59]. Since external plasticisers such as phthalate esters are not chemically bonded to the PVC polymer but are present as mobile constituents within the plastic matrix, they tend to migrate to the surface and the surrounding environment [57,60]. Accumulation of a plasticiser at the surface occurs when the evaporation rate of the plasticiser from the surface into the surrounding air is slower than its migration from the bulk plastic to the surface. This is the standard mechanism discussed for PVC plasticised with high molecular weight phthalates like diethyl hexyl phthalate (DEHP). The resulting visible accumulation can be described as ‘sweating’ and can manifest as tacky-to-the-touch surfaces [61].

**Figure 7:**
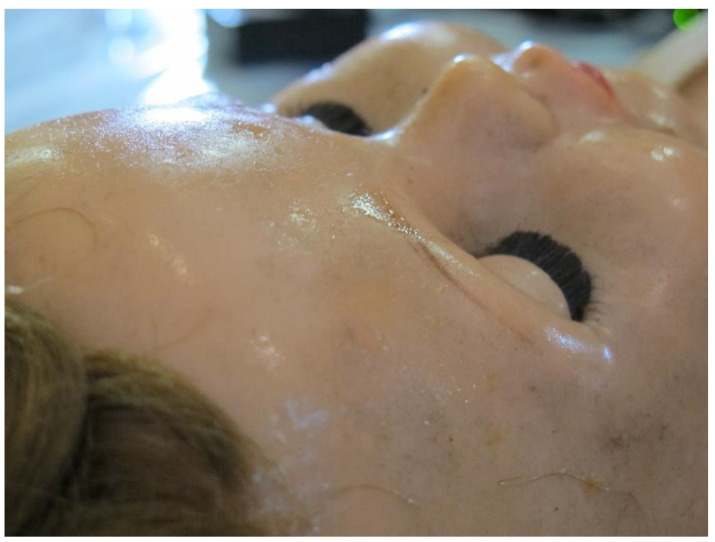
Plasticiser migration on PVC head of doll K2 (with CA body, arms and legs). A characteristic ‘sweaty’ surface with trapped surface dirt is visible. Image ©Tate.

Tacky surfaces were noted on the PVC heads of CA dolls E6 and K2 (the latter shown in Figure 7). Samples were taken of liquid exudate on the surface of the PVC heads and PyGCMS confirmed the presence of diethyl hexyl phthalate (DEHP) the most commonly used plasticiser for PVC. Plasticiser migration can also cause PVC moulded structures to deform and distort, although this has not yet occurred for dolls in this artwork. Some of the PVC elements with more significant plasticiser migration also had an associated strong smell. The migrated plasticiser and resulting tacky surfaces had also resulted in noticeable surface dirt retention. Continued dirt adhesion over time on tacky surfaces with exuded plasticisers could begin to appear disfiguring, and may contribute toward further degradation of the plastic [62]. Dry cleaning could be carried out to remove exuded plasticiser and associated surface dirt [63], or if dry cleaning is found to be ineffective, the use of carefully selected solvents chosen to minimise risks of plasticiser leaching could be explored for soiling removal [62]. However, prior to considering any treatment, it would be necessary to discuss surface cleaning with the artist since she values the worn and used appearance of the dolls (see Section 3.5).

**Figure 8:**
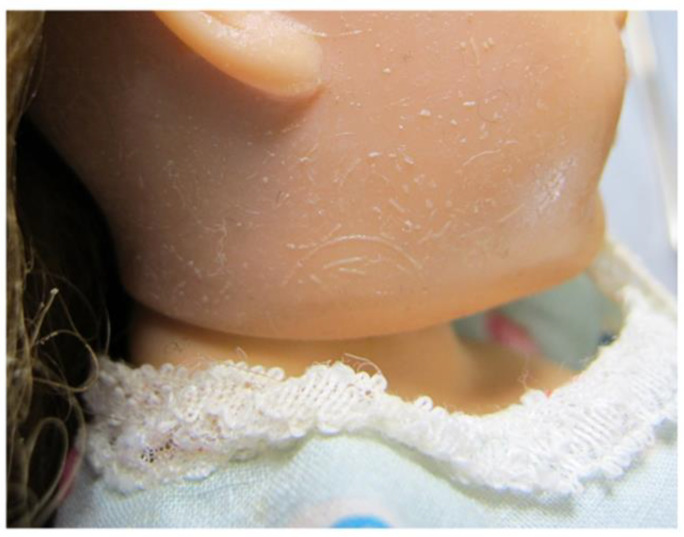
White surface crystals, observed on the PVC head of doll A8, which had a CA body. Image ©Tate.

**Figure 9:**
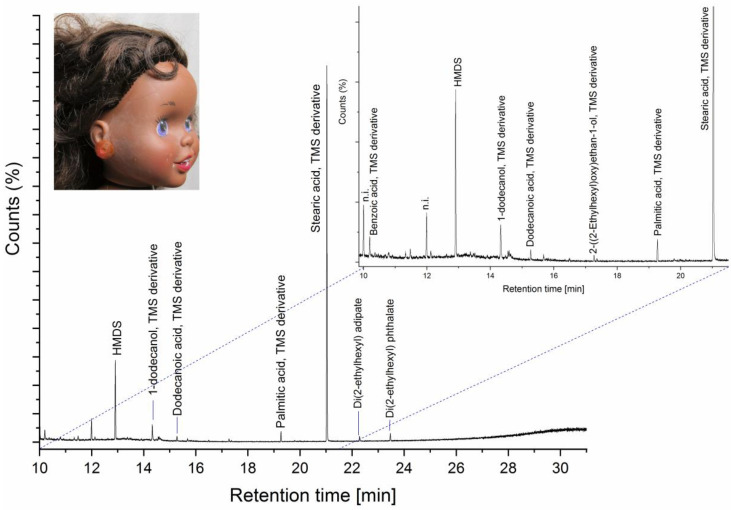
Detail of the PVC head of doll E4 showing a white surface bloom (image insert, ©Tate.) and HMDS-PyGCMS pyrogram of a sample of the white bloom. The TMS derivative of stearic acid dominated the sample, and small amounts of the TMS derivatives of 1-dodecanol, dodecanoic acid, palmitic acid were also identified, along with traces of di(2-ethylhexyl)adipate (DEHA) and di(2-ethylhexyl) phthalate (DEHP).

White efflorescence was also seen on several PVC dolls. Distinct white surface crystals were observed on some of the PVC heads, e.g., on the head of doll A8 (Figure 8). PyGCMS of a polymer sample (S.I., Figure S8 and Table S6) taken from the head of doll A8 identified di(2-ethylhexyl) phthalate (DEHP) and major amounts of cyclopentanone, which is a pyrolysis product of adipic acid (hexanedioic acid) [64]. Esters of adipic and phthalic acid are used in combination [65] as external plasticisers for PVC, with DEHP being one of the most widely used phthalate plasticisers [66]. The white surface crystals shown in Figure 8 were not analysed, however hydrolysis of phthalate plasticisers and formation of phthalic acid crystals have been reported for degraded PVC [8]. A more distributed white bloom was seen on the PVC head of doll A1 (Figure 9). HMDS-PyGCMS of the white bloom identified the trimethyl silyl (TMS) derivatives of stearic acid, which dominated the sample; small amounts of the TMS-derivatives of dodecanoic acid, 1-dodecanol and palmitic acid, as well as traces of di(2-ethylhexyl)adipate (DEHA) and di(2-ethylhexyl) phthalate (DEHP) plasticisers. Carboxylic acids with a carbon chain length ≥ 12 and 1-dodecanol are heat stabilisers or lubricants that are used for PVC, and certainly stearic acid is commonly used, up to around 3% by weight in PVC [67,68].

**Figure 10:**
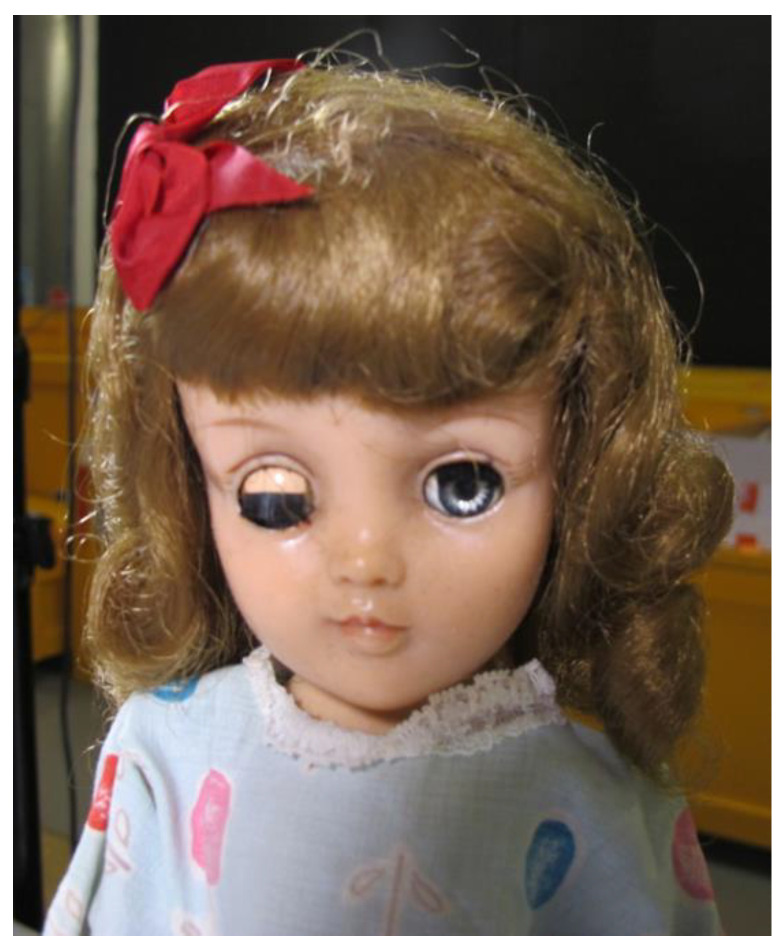
Failed sleep eye mechanism in CA doll A8 with a PVC head. Image ©Tate.

Discolouration of PVC was often seen across the dolls, which may be the result of dehydrochlorination [69] and formation of polyenes [57]. Observed colour changes included yellowing (e.g., Figure 6c, head) and whitening (e.g., Figure 6d, head and arms). Whilst discolouration of PVC to brown and black has been reported [8,57] no such changes were observed across this group of dolls. In general PVC discolouration was most evident on the older dolls and particularly those with bodies and limbs made from CA. Finally, several dolls with PVC heads featured eyes that did not fully open (e.g., Figure 10 below), which, as discussed in the introduction, could be due to trapped and/or corroded eye mechanisms [14,15].

#### 3.4.5. Polystyrene Dolls

PS was produced commercially from 1937 [8]. PS was identified in 11 dolls dated from the 1970s to the 1980s. Often torsos had been made with PS with other plastics such as PE or PVC used for arms and legs, and other synthetic fibres for hair and clothing (S.I., Table S1). PS is vulnerable to light exposure and photoxidation [8], and some of the PS components of the dolls appear to have started to yellow (e.g., Figure 6b, torso of doll I8; Figure 6e,f), although the presence of colourants was not investigated. The dolls with PS components tended to be both younger, and aside from any yellowing in good condition (Table 6) relative to dolls made with other materials and older dolls. Other companion components, e.g., any associated PVC heads, were similarly in a less advanced state of degradation compared to those of the older (CA) dolls (see Section 3.4.3 and Section 3.4.4).

**Figure 11:**
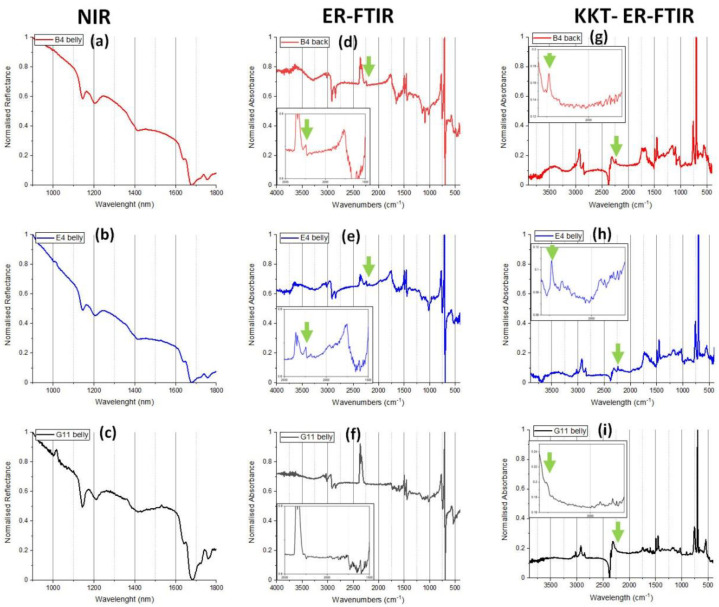
ABS was tentatively identified in dolls E4, B4 and G11 using NIR (**a**–**c**). NIR spectra were compared to ER-FTIR spectra (**d**–**f**) and KK transformed ER-FTIR spectra (**g**–**i**) to distinguish between PS and possible ABS. Where a nitrile band was visible in ER-FTIR spectra at ~2250 cm^−1^ (as indicated with green arrows) ABS was identified. For doll G11, there was only a weak indication of a nitrile band observed in ER-FTIR spectra (**i**).

The difficulty of correctly distinguishing between closely similar polymers, such as ABS and PS using NIR spectroscopy has been reported [70]. In this study NIR was used to distinguish tentatively between ABS and PS in three dolls (E4, B4, and G11). Examination of ER-FTIR, and KK transformed ER-FTIR spectra (Figure 11) and comparison to reference spectra (S.I., Figure S9) provided more evidence for ABS attribution in dolls E4 and B4 due to the presence of nitrile bands visible at ~2250 cm^−1^. In the case of doll G11, there was only a very weak indication for a nitrile band however due to spectral noise, this was not particularly clear (Figure 11i). A carbonyl absorption at ~1750 cm^−1^ was visible in ER-FTIR spectra of dolls B4 and E4 (Figure 11d,e) which corresponded to a broad band with maxima at ~1750 cm^−1^ in KKT-FTIR spectra (Figure 11g,h). The presence of a carbonyl band could indicate photoxidation of the suspected ABS [71,72,73] and/or the presence of additives, but further investigation would be required to investigate this further. Doll G11 did not present a carbonyl band at 1750 cm^−1^ (Figure 11f,i). To be more certain of the identification of ABS, further analysis would be required (e.g., using PyGCMS, which would necessitate sampling).

#### 3.4.6. Clothing and Hair

The identification of clothing and hair fibres associated with the dolls was a lower priority for the purposes of this survey than identifying the main polymers used for body and limbs, which are structurally more significant. Samples were taken from fibres used for hair in only a limited number of cases (S.I. Table S1) to identify materials that were showing obvious signs of degradation. Wool was identified as the matted hair for doll J4, a composition doll. Synthetic fibres made of polypropylene were presenting significant fragmentation in dolls G1 and H1, and brittle and friable polyamide fibres were identified as the hair of doll B11 (for FTIR spectra see S.I., Figure S10).

### 3.5. Implications of the Condition and Technical Survey

After reviewing the analytical survey results and the in-depth condition reporting of the 44 dolls, the information gathered was collated with the broader condition survey of all 162 dolls. This process facilitated the identification of a group of 11 dolls considered to be at high risk (i.e., in poor condition according to Table 1) at the time of condition checking. These dolls are fragile and extremely vulnerable to the degree of handling and movement required during installation and deinstallation, which could promote the formation or propagation of cracks/breaks.

Of the 11 high-risk dolls, six were identified as composition dolls (D11, I3, I10, J4, K7 and L9). All of these dolls showed severe cracking of applied paint layers and the underlying moulded material forming the structure of the dolls was fragile (see S.I., Figure S11). Two of the composition dolls had losses in their feet most likely caused by the hot glue mounting method used in the past. Two of the dolls classed as high risk (K2 and C13, shown in Figure 4a and Figure 4b, respectively) had heavily degraded CA parts with surface crystals caused by migrated plasticiser, alongside multiple cracks and losses (see S.I., Figure S12). CA doll K2 also had a PVC head with a weeping, sticky surface, caused by migrated phthalate plasticisers. Two high-risk dolls had PS legs and torsos (F8 and G11) with significantly cracked and discoloured PE components (see S.I., Figure S13). A heavily crazed nitrocellulose paint layer with many losses was additionally present on doll G11. Finally, Doll I14, which did not undergo materials analysis but was categorised as high risk, had large cracks of the legs (likely PE), with yellowing of the fingers (likely PVC). Devising tailored mounting methods for storage and display may reduce the risk of mechanical damage. However, some of the high-risk dolls may already be too physically vulnerable to be re-displayed as they cannot stand upright as the artist intends. For these, a discussion with the artist is required to understand what conservation treatment is appropriate.

In addition to the high-risk dolls, 11 more dolls were placed on a ‘watch list’. These dolls can safely be displayed in a standing position but were classed as being in a fair condition according to the definition in Table 1. These dolls were considered likely to fall into the ‘high risk’ category in the near future. Particular attention will be given to dolls on the watch list prior to each installation/de-installation. This included: one composition doll (B6); four dolls with identified CA or PVC parts (A8, E6, I12, L13) with signs of active degradation, e.g., embrittlement or the presence of crystalline or liquid exudates; two PE dolls (B10 and H7) with discolouration and surface deposits; one PS doll (K11) showing embrittlement. Also included were four dolls which were not analysed (F1, F3, G1 and L4) but showed evidence for embrittlement and possible plasticiser migration in addition to stiff or embrittled hair.

#### 3.5.1. Implications for Display

A significant, obvious, concern for the ongoing preservation of this artwork is the risk posed by the current mounting method. As discussed earlier, thus far when arranged for display, the dolls have been mounted upright, by gluing their feet to the gallery floor using a hot-melt adhesive. PyGCMS analysis (S.I., Figure S14) indicated that the adhesive used in the past was an ethylene/vinyl acetate (EVA) copolymer. This adhesive is not easily reversible and repeated past application to the dolls’ feet, and detaching of the feet from the gallery floor, as noted in Section 3.1.5, has caused breaks or losses in 113 of the 162 dolls. The survey highlighted that due to loose joints or textile bodies, not all of the dolls now have the rigidity to stand upright with a method that only secures the dolls at their feet. Further work includes investigating alternative methods for mounting the dolls, particularly for those dolls identified as fragile or brittle, while ensuring that any mounting method remains discreet.

#### 3.5.2. Implications for Storage

Upon acquisition, the dolls arrived each enclosed within polyester bags, within lightly padded cardboard boxes, collectively stored and transported within a foam-lined wooden artwork case. The artwork has been stored within Tate’s climate-controlled storage spaces since arrival. The current storage method prevents exposure to light, which is a major factor in the degradation of all plastics. However, due to the range of polymer types present in these dolls and the challenges inherent to each plastic type, bespoke storage solutions may need to be developed for the artwork and/or specific components therein to slow down further degradation. In this case, the analytical work has been highly informative as plastic identification forms the basis for amended storage strategies. For example, CA components may benefit from the use of packaging which absorbs acidic vapours [8]. In contrast, for plasticised PVC, reducing plasticiser loss that occurs by evaporation is generally considered advantageous [57]. Avoiding storage of PVC objects in open environments and using wrappings that do not promote plasticiser loss (e.g., silk, paper [74]), may help reduce the rate of plasticiser loss from PVC. Due to the heterogenous nature of the dolls, it will be challenging to find a single storage solution where conflicting ideal storage conditions are present; hence, compromises will need to be made for any overall packing solution. Cool or cold storage is known to slow down the degradation of most plastics [9,75] if done with care with respect to risks around contraction and stiffening under colder temperatures [8]. However, given the size and scale of this artwork this is unlikely to be a feasible solution, from a practical, environmental, and financial perspective.

In this case, the original card box packing may be advantageous since it offers a modular system where each box can be tailored to the needs of each doll. This might include the use of materials such as charcoal cloth or acid-free tissue and card to absorb acidic off-gassing from CA components or additional non-stick wrapping materials for dolls with sticky surfaces, such as PVC dolls with migrated plasticisers, which may otherwise become stuck to packing materials. The modular system also means dolls can be easily accessed for condition checking, or treatment, and boxes can easily be replaced as needed. One improvement that is needed for all dolls, is a level of physical support within the boxes to prevent movement from vibrations or shock during transport, and to prevent compression of vulnerable components. For example, 62 of the dolls had matted and compressed hair. Although some of this may be due to past use and the dolls being repeatedly laid flat on their backs, this is exacerbated by the continued storage of the dolls in this conformation. It is expected that the foam lining within the packing case in which the dolls are stored, will absorb some level of vibration and shock that may be experienced during transport, however further shock absorption, such as using conservation grade closed-cell PE foam, could be added within the boxes to further limit shock and vibration.

## 4. Conclusions

The collection of 162 dolls in *Zoe Leonard’s Mouth Open Teeth Showing* encompasses many synthetic plastics encountered in heritage collections and exemplify several characteristic types of degradation. A representative sub-set of 44 dolls was selected for materials characterisation, and this, in conjunction with the detailed condition surveys, has informed the attribution of likely polymer classes for the wider set of dolls. The non-destructive spectroscopic methods utilised (ER-FTIR and NIR) typically enabled identification of the main polymer(s) present, with the aid of a in-house polymer library. However, of the 17 dolls that were only analysed using ER-FTIR and NIR, the polymeric materials present in three dolls (F3, H11 and J6, see: Table S1, S.I.) could not be identified. This was due to the use of non-reflecting dark plastic in doll J11, and the presence of additional bands in ER-FTIR spectra which were not present in available reference spectra, thus highlighting the need for developing accessible, extensive external reflectance infrared libraries to aid data interpretation. For surfaces not amenable to characterisation using reflectance IR techniques, for the detailed identification of additives (e.g., plasticisers), and for more complex or unusual polymeric materials, appropriate mass spectrometric techniques were required.

This combined condition and analytical survey enabled the documentation of a series of specific ageing phenomena associated with the varied materials found in this unique collection of dolls. Key ageing phenomena identified included colour change, soiling, exudation of plasticisers, paint delamination, physical damage, matted hair and eyes that no longer fully open. The information gained will serve to inform future discussions with the artist where her thoughts on the various examples of degradation phenomena will be explored. Determining which manifestations of age and change the artist considers intrinsic to (the meaning of) the work will help inform appropriate non-interventions in the case of dirty faces, torn clothing or matted hair, and interventions in the case of mounting and long-term storage. This would help balance the ongoing needs of preservation with the artist’s wishes relating to the ongoing lives of the dolls as they each move from being individually loved objects to a group of dolls with a new message and meaning. It would also be helpful to explore installation parameters, e.g., discussing alternative solutions for mounting the dolls in an upright position and considering the range of lux levels for the artwork whilst on display. It will also be crucial to define when it may become necessary to replace individual dolls, and guidance as to if, how, and when this may be carried out.

## Figures and Tables

**Table 1:** Vulnerability scale used to assess condition, drawing on condition definitions described by Keneghan [17] the BMT and the National Trust.

Condition	Numerical Score	Description
Excellent	1	Little or no evident damage.
Good	2	Minor amount of damage and/or loss of original and added material, or with light discoloration or accretions.
Fair	3	Noticeable damage and loss, and appears disfigured with visible accretions.
Poor	4	Considerable and/or significant loss of original or added material or major damage/breakage or disfigurement. May be endangering other objects and surfaces.

**Table 2:** Summary of condition of the dolls identified as having CA components.

Doll Number		A8	C13			E6		E12		K2	
Date		1950s	1971			1950s		?		?	
Body Part		Body	Body	Arms	Legs	Body	Arms	Head	Legs	Arms	Legs
**Observations**	Good condition *	✓	-	-	-	✓	✓	-	✓	-	-
	Dirt/staining	-	✓	-	✓	✓	✓	✓	✓	✓	✓
	Minor scratches/abrasions	-	✓	-	✓	✓	✓	✓	✓	✓	✓
	Cracking/ embrittlement	-	✓	✓	-	-	-	-	-	✓	✓
	Discolouration	-	-	-	-	-	-	-	-	-	-
	Surface bloom or crystalline bloom	-	✓	✓	✓	-	-	-	-	✓	✓
	weeping/sweating	-	-	-	-	-	-	✓	-	-	-
	Sticky exudate	-	-	-	-	-	-	✓	-	-	-
	Strong smell	-	✓	✓	✓	✓	✓	-	-	✓	✓
	Striations on surface of plastic	-	-	-	-	-	-	-	-	-	-
	Moulding lines visible	-	✓	✓	✓	-	-	-	-	-	-
	Rubbery feel	-	-	-	-	-	-	-	-	-	-
	Feels plasticised	-	-	-	-	-	-	-	-	-	-
	Rigid	-	-	-	-	-	-	-	✓	✓	-

* See definitions in Table 1.

**Table 3:** Summary of condition of the dolls identified as having PE components.

Doll Number		A1	B10		B11	D5			E14		G3	G8		H1		H7		I1	I4			I5	J8	K3	L11	
Date		1960s	1970s		1960s	1950s			1950s		1960s	?		1967		1961		1960s	?			1960s–70s	1950s–60s	?	1950s–60s	
Body Part		Legs	Body	Legs	Legs	Body	Arms	Legs	Legs	Body	Body	Body	Legs	Body	Legs	Body	Legs	Body	Body	Legs	Other	Body	Body	Legs	Body	Legs
**Observations**	Good condition *	✓	✓	-	✓	✓	✓	✓	-	-	✓	✓	✓	✓	✓	✓	✓	✓	✓	✓	✓	✓	✓	✓	✓	✓
	Dirt/staining	-	✓	✓	-	-	-	-	✓	✓	✓	✓	✓	-	-	✓	✓	✓	-	✓	✓	✓	✓	✓	-	✓
	Minor scratches/ abrasions	✓	✓	✓	-	-	-	-	✓	✓	✓	-	-	✓	✓	✓	✓	✓	-	✓	✓	✓	✓	✓	-	-
	Cracking/ embrittlement	-	-	-	-	-	-	-	✓	✓	-	-	-	-	-	-	-	-	-	-	-	-	-	-	-	-
	Discolouration	-	-	✓	✓	-	-	-	-	-	-	-	-	-	-	-	-	-	-	-	-	-	-	-	-	-
	Surface bloom or crystalline bloom	-	-	-	-	-	-	-	-	-	-	-	-	-	-	-	-	-	-	-	-	-	-	-	-	-
	Weeping/sweating	-	-	-	-	-	-	-	-	-	-	-	-	-	-	-	-	-	-	-	-	-	-	-	-	-
	Sticky exudate	-	-	✓	-	-	-	-	-	-	-	-	-	-	-	-	-	-	-	-	-	-	-	-	-	-
	Strong smell	-	-	-	-	-	-	-	-	-	-	-	-	-	-	✓	✓	-	-	-	-	-	-	-	-	-
	Striations on surface of plastic	-	-	-	-	-	-	-	✓	-	-	-	-	-	-	-	-	-	✓	-	-	✓	-	-	-	-
	Moulding lines visible	-	-	-	-	-	✓	✓	-	-	-	-	-	✓	✓	✓	✓	✓	-	-	-	-	✓	✓	-	-
	Rubbery feel	-	-	-	-	-	-	-	-	-	-	-	-	-	-	-	-	-	-	-	-	-	-	-	-	-
	Feels plasticised	-	-	-	-	-	-	-	-	-	-	-	-	-	-	-	-	-	-	-	-	-	-	-	-	-
	Rigid	-	-	-	-	-	✓	✓	✓	✓	-	-	-	✓	✓	✓	✓	✓	-	-	-	-	✓	✓	-	-

* See definitions in Table 1.

**Table 4:** Summary of FTIR results, and elemental composition via EDX of samples identified as PVC. Samples were taken from 11 dolls.

Doll, Sample Number	Description	Results of FTIR Analysis	C	O	Cl	Al	S	Ca	Si	Fe	P	Na	Ti	Mg	Cd	Zn	K	Cu	Mo	Ba
A1, S2	Plastic used for left arm	Phthalate plasticiser	✓	✓	✓	✓	✓	✓	-	✓	-	✓	-	-	✓	-	-	-	-	✓
A8, S1	Flexible plastic used for head	Phthalate plasticiser	✓	✓	✓	✓	✓	-	✓	-	✓	-	-	-	-	-	-	-	✓	-
D10, S1	Plastic used for head	Phthalate plasticiser, chalk possible calcium oxalate	✓	✓	✓	✓	✓	-	-	-	-	✓	✓	-	-	✓		-	-	-
E6, S1	Flexible plastic used for head	Phthalate plasticiser, chalk	✓	✓	✓	✓	✓	-	✓	✓	✓	-	✓	-	-	-	✓	-	-	-
H1, S3	Flexible plastic used for head	Phthalate plasticiser, chalk	✓	✓	✓	✓	✓	✓	✓	✓	✓	✓	-	✓	-	-	✓	-	-	-
H5, S1	Little finger of left hand	Phthalate plasticiser	✓	✓	✓	✓	✓	✓	✓	-	-	✓	-	✓	-	-	-	-	-	-
H7, S2	Pale discoloured, right arm plastic	Phthalate plasticiser, chalk	✓	✓	✓	✓	✓	✓	✓	-	✓	✓	-	✓	-	-	-	✓	-	-
J8, S2	Right arm plastic	Phthalate plasticiser	✓	✓	✓	✓	✓	-	✓	-	✓	✓	-	✓	-	-	-	-	-	-
K14, S1	Pink flexible transparent plastic with sticky exudate. Used for bag strap accessory on doll.	Phthalate plasticiser	✓	✓	✓	✓	✓	-	✓	-	-	-	-	-	-	-	-	-	-	-
K2, S3	Flexible degraded material used to make head. Exudate visible.	Phthalate plasticiser	✓	✓	✓	✓	✓	✓	✓	✓	✓	✓	✓	✓	-	-	-	-	-	-
L13, S2	Lighter pink flexible plastic from trunks of doll	Phthalate plasticiser titanium white	✓	✓	✓	✓	✓	✓	✓	✓	-	-	✓	-	-	-	-	-	-	-

**Table 5:** Summary of condition of the dolls identified as having PVC components. Dolls with additional CA components are identified.

Doll Number		A1	A8	D10	E6	H1	H5	H7	J8	K14	K2		L13
Date		1960s	1950s	1950s	1950s	1967	1971	1961	1950s–60s	1985	?		1959
Body Part		Arms	Head	Head	Accessory	Head	Head	Arms	Arms	Arms	Accessory	Head	Body
**Observations**	Good condition *	-	-	-	-	-	✓	✓	-	✓	-	-	✓
	Dirt/staining	✓	✓	-	✓	✓	-	-	✓	✓	-	✓	✓
	Minor scratches/abrasions	✓	-	✓	✓	-	-	-	✓	✓	-	-	✓
	Cracking/ embrittlement	-	-	-	-	-	-	-	-	-	-	-	-
	Discolouration	✓		✓	✓	✓	-	-	✓	-	-	✓	
	Surface bloom or crystalline bloom	-	✓	-	-	-	-	-	✓	-	-	-	-
	weeping/sweating	-	✓	-	-	-	-	-	-	-	-	✓	-
	Sticky exudate	-	-	-	-	✓	-	-	-	-	✓	✓	✓
	Strong smell	-	-	-	-	-	-	-	✓	-	-	-	
	Striations on surface of plastic	-	-	-	-	-	-	-	-	-	-	-	-
	Moulding lines visible	-	-	-	-	-	-	-	-	-	-	-	-
	Rubbery feel	-	-	-	-	-	✓	-	-	-	-	-	-
	Feels plasticised	-	-	-	-	✓	✓	-	-	-	✓	✓	-
	Rigid	-	-	-	-	-	-	-	-	-	-	-	-

* See definitions in Table 1.

**Table 6:** Summary of condition of the dolls identified as having PS components.

Doll Number		B7	B11	C14	F8		G11		H5	I8	J7		K11
Date		1998	1960s	1989	1977		1960s		1971	1980	1985		1970s
Body Part		Body	Body	Body	Body	Legs	Body	Legs	Body	Body	Body	Arms	Body
**Observations**	Good condition *	✓	✓	✓	-	✓	✓	-	✓	-	✓	✓	✓
	Dirt/staining	-	-	-	✓	-	✓	✓	-	-	✓	✓	✓
	Minor scratches/abrasions	-	-	-	✓	-	-	-	-	✓	✓	✓	-
	Cracking/embrittlement	-	-	-	✓	-	-	✓	-	-	-	-	-
	Discolouration	-	-	-	✓	-	-	✓	-	✓	-	-	✓
	Surface bloom or crystalline bloom	-	-	-	-	-	-	-	-	-	-	-	-
	weeping/sweating	-	-	-	✓	-	-	-	-	-	-	-	-
	Sticky exudate	-	-	-	-	-	-	-	-	-	-	-	-
	Strong smell	-	-	-	-	-	-	-	-	-	-	-	-
	Striations on surface of plastic	-	-	-	-	-	-	-	-	-	-	-	-
	Moulding lines visible	-	-	-	✓	✓	✓	-	-	-	-	-	-
	Rubbery feel	-	-	-	-	-	-	-	-	-	-	-	-
	Feels plasticised	-	-	-	-	-	✓	-	-	-	-	-	-
	Rigid	-	-	✓	✓	✓	-	✓	✓	-	✓	-	✓

* See definitions in Table 1.

## Data Availability

The data presented in this study are available on request from the corresponding author.

## Supplementary Materials

The following supporting information can be downloaded at: https://www.mdpi.com/article/10.3390/polym15010034/s1, S.I File 1, which includes: Figure S1: The artist labelled each doll with a letter and number, and provided a layout plan and instructions for displaying the dolls. This diagram shows the display layout of the 162 dolls comprising the artwork); Figure S2: An example of a maker’s mark visible on doll H1, which reads “UNEEDA DOLL 19©67, MADE IN ITALY, 20”; Table S1: Summary of the 44 selected dolls, along with their makers’ marks (where present), estimated or known dates, and the analytical techniques employed for each doll; Table S2: Summary of FTIR and PyGCMS analysis of selected samples; Figure S3: Normalised transmission FTIR analysis of a bulk composition sample from a damage of doll I10; Figure S4: Normalised transmission FTIR and EDX spectrum (shown as insert) of the nitrocellulose pink layer of paint on doll I10; Table S3: Band assignments for FTIR spectra shown in Figures S3 and S4; Figure S5 Normalised transmission FTIR spectra of samples taken from cellulose acetate doll C13 (a) cellulose acetate sample from edge of sound hole, which appeared to be in a good condition; (b) cellulose acetate from degraded hip area with powdery and greyed surface; (c) acicular crystalline efflorescence from hand; Table S4: Band assignments for the IR spectra presented in Figure S5 above; Figure S6: PyGCMS anlaysis of hip plastic from CA doll C13. TPP and DEP plasticisers were identified; Table S5. Peak assignments for the pyrogram shown in Figure S6; Figure S7: 3D Scatter plot of PCA analysis of various dolls analysed using NIR; Figure S8: PyGCMS analysis of a sample of the flexible plastic used to construct the head of doll A8. Dominant pyrolysates were cyclopentanone, and di(2-ethylhexyl)phthalate; Table S6. Peak assignments for the pyrogram shown in Figure S8; Figure S9: Comparison of ER-FTIR spectra of doll E4, to ER-FTIR reference spectra for PS and ABS. The ER-FTIR spectra of PS and ABS appear closely similar, however a nitrile band at ~2250 cm−1 helps distinguish between the two polymers.; Figure S10: Normalised transmission FTIR spectra of hair fibres from four dolls: (a) matted woollen hair fibres of doll J4; (b) Friable and fragmenting PP hair fibres from doll H1; (c) Friable and fragmenting polyamide fibres from doll B11; (d) Friable and fragmenting PP hair fibres from doll G1; Figure S11: Details of composition doll D11 showing cracking and lifting paint surface, and multiple losses (a) head with lifting paint (b) missing finger tips showing the underlying brown material that forms the shape of the doll. (c) similarly to b, only missing a foot; Figure S12: Details of doll K2 showing (a) sweating of the PVC head (b) cracking and loss to the CA arm; Figure S13: (a) Details of dolls G11 PS leg with flaking NC paint layer (b) Doll I14, showing cracking of (likely PE) legs which may worsen during handling and display. (c) crystal deposits on surface of arm, S.I. File 2, an Excel spreadsheet containing ER-FTIR data of reference plastic standards used to aid data interpretation.
