# Supplementary material for: Exploring the Materials and Condition of 20th-Century Dolls in Zoe Leonard’s Mouth Open, Teeth Showing 2000
Source: Polymers (Basel). 2022 Dec 22;15(1):34. doi: 10.3390/polym15010034 (PMC9824361; doi:10.3390/polym15010034)
Supplement: Supplementary file 1 [file polymers-15-00034-s001.zip › polymers-2080263-S.I. File 1.pdf]

Supplementary Information File 1 (S.I.)

Exploring the materials and condition of 20th-century dolls in Zoe Leonard's *Mouth Open, Teeth Showing* 2000

Judith Lee[1]*, Libby Ireland[1], Joyce H. Townsend[1], Bronwyn Ann Ormsby[1], Angelica Bartoletti[1], Deborah Cane[1], Simoní Da Ros[2], Rose King[2], Isabella del Gaudio[2] and Katherine Curran[2]

1.Conservation Department, Tate Britain, Millbank, London SW1P 4RG, UK
2.UCL Institute for Sustainable Heritage, University College London, 14 Upper Woburn Place, London WC1H 0NN, UK

List of Figures and Tables **Page Number**

Figure S1	2
Figure S2	3
Figure S3	10
Figure S4	11
Figure S5	13
Figure S6	14
Figure S7	15
Figure S8	16
Figure S9	17
Figure S10	18
Figure S11	19
Figure S12	19
Figure S13	19
Table S1	4-6
Table S2	7-9
Table S3	12
Table S4	13
Table S5	14
Table S6	16

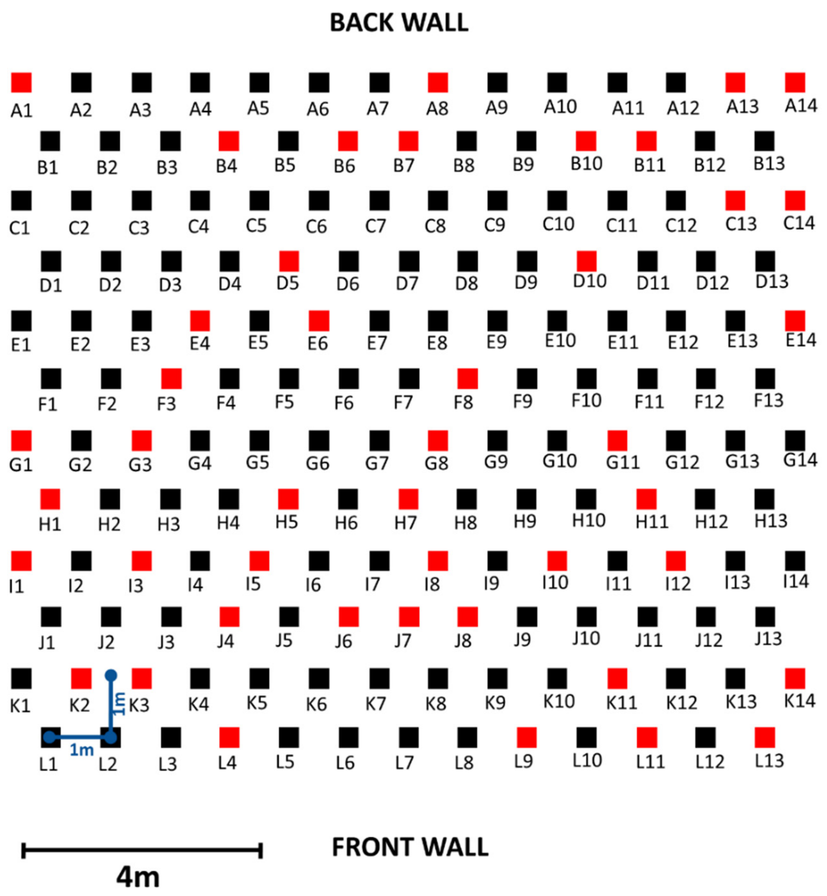

Figure S1. The artist labelled each doll with a letter and number, and provided a layout plan and instructions for displaying the dolls. This diagram shows the display layout of the 162 dolls comprising the artwork. Each doll is represented by a square and is identified by a letter and a number combination. There is a 1m spacing between each doll when on display (see annotations in blue). The 44 dolls selected for materials analysis are shown in red.

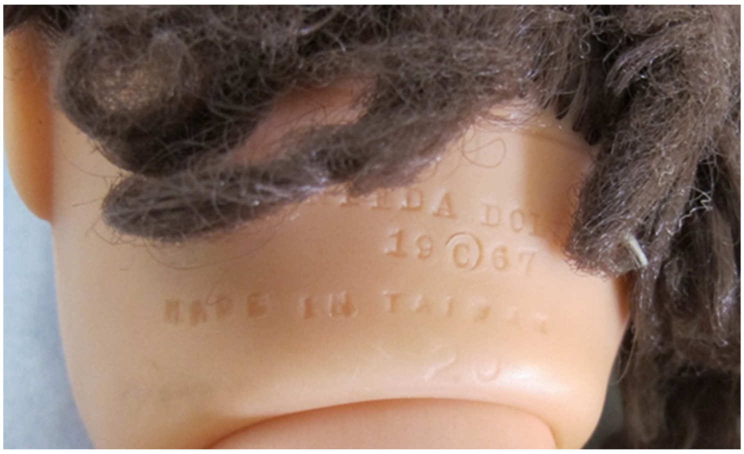

Figure S2. An example of a maker’s mark visible on doll H1, which reads “UNEEDA DOLL 19©67, MADE IN ITALY, 20”. Image ©Tate.

Table S1. Summary of the 44 selected dolls, along with their makers' marks (where present), estimated or known dates, and the analytical techniques employed for each doll. The doll number gives the position of each doll when displayed (see Fig. S1). Polymers identified include: PE = polyethylene, PP = polypropylene, PS = polystyrene, CA = cellulose acetate, CN = cellulose nitrate, PVC = polyvinyl chloride, PA = polyamide.

**Doll number**	**Transcription of makers' marks where present** *All located on back of heads unless stated otherwise. n.i = none identified.*	**Date** *estimated dates as decades, exact years based on makers' marks or identified dolls*	**Analysis technique used** (*✓ = specified technique used; blank = not used)*					**Polymers identified for main structural elements (head and/or body)**	**Polymers identified for accessories, clothing or hair (limited sampling)**
			**ER-FTIR**	**NIR**	**FTIR**	**EDX**	**PyGCMS**		
A1	Regal Toy Made In Canada	1960s	✓	✓	✓			PE, PVC	
A8	MK-14, P90 Ideal Doll	1950s	✓	✓	✓		✓	CA, PVC	
B4	MATTEL. INC. 1988, China	1988	✓	✓				ABS, PVC	
B6	A.C (? American Character Doll (1919-1968) [6])	1960s	✓	✓				CA	
B7	© 1998 DSI	1998	✓	✓	✓			PS	
B10	Ozen, Japan Pat 931179 US. Pat.3467393	1969	✓	✓				PE	
B11	Made in Italy	1950s	✓	✓	✓			PS, PE	PA
C13	Horsman Dolls Inc.	1971	✓	✓	✓	✓	✓	CA	
C14	U.S. Patent No. 4.801.286, MADE IN CHINA	1988	✓	✓				PS	
D5	n.i	1950s	✓	✓				PE	
D10	n.i	1950s	✓	✓	✓	✓		PVC	
E4	©1996 TYCO Ind. INC. Made in China	1996	✓	✓	✓			ABS, PVC	
E6	210 UNEEDA	1950s-60s	✓	✓	✓		✓	CA, PVC	
E14	EEGEE	1950s-60s	✓	✓	✓			PE	

Doll number	Transcription of makers' marks where present *All located on back of heads unless stated otherwise. n.i = none identified.*	Date *estimated dates as decades, exact years based on makers' marks or identified dolls*	Analysis technique used (✓ = specified technique used; blank = not used)					Polymers identified for main structural elements (head and/or body)	Polymers identified for accessories, clothing or hair (limited sampling)
			ER-FTIR	NIR	FTIR	EDX	PyGCMS		
F3	P-91 Ideal Doll, Made in U.S.A	1950s	✓	✓				Not identified	
F8	IDEAL TOY CORP T-16G-H-276	1977	✓	✓				PS	
G1	© 1998 TRU MADE IN CHINA P M419-B; Inscription on back of arm reads: 'PM416'	1998	✓	✓	✓			Not identified	PP
G3	Hong Kong	1960s-70s	✓	✓				PE, PVC	
G8	n.i	?	✓	✓				PE, PVC	
G11	GeGe, Brevete BD3M	1968	✓	✓	✓			PS, NC paint	
H1	UNEEDA DOLL CO, 1967 ©, Made in Taiwan, 20	1967	✓	✓	✓	✓	✓	PE, PVC	PP
H5	Ideal Toy Corp, HK-18	1971	✓	✓	✓			PE, PS, PVC	
H7	EFFANBEE© 1961	1961	✓	✓	✓	✓	✓	PE, PVC	
H11	©Ideal Toy Corp, 1978, B-66	1978	✓	✓				PVC	
I1	UNEEDA 2	1960s	✓	✓				PE, PVC	
I3	n.i	1940s?	✓	✓	✓			Composition	
I5	Made In China	1960s-1970s	✓	✓				PE	
I8	Italy, Migliorati Inscription on back reads: 'Made in China'.	1960s	✓	✓				PS, PE	
I10	n.i	1940s			✓			Composition, NC paint	
I12	n.i	?	✓	✓	✓	✓		CA	

**Doll number**	**Transcription of makers' marks where present** *All located on back of heads unless stated otherwise. n.i = none identified.*	**Date** *estimated dates as decades, exact years based on makers' marks or identified dolls*	**Analysis technique used** *(✓ = specified technique used; blank = not used)*					**Polymers identified for main structural elements (head and/or body)**	**Polymers identified for accessories, clothing or hair (limited sampling)**
			**ER-FTIR**	**NIR**	**FTIR**	**EDX**	**PyGCMS**		
J4	n.i	?			✓	✓		Composition, NC paint	Wool
J6	n.i	?	✓	✓		✓		Not identified	
J7	©198? Panosh Place 15 Inscription on back of doll reads: '1985 Panosh Place, China'	1985	✓	✓	✓	✓		PS	
J8	Reliable, Canada	1960s	✓	✓	✓	✓		PE, PVC	
K2	X	No older than 1927	✓	✓	✓	✓	✓	CA, PVC	
K3	Inscription on back of torso reads: 6B(8?) Made in France	1960s	✓	✓	✓	✓	✓	PE, NC paint	
K11	n.i		✓	✓				PS, PVC	
K14	©1988 Panache Peach?', 28"	1988	✓	✓		✓	✓	PVC	
L4	Furga Italy	1960s-70s	✓					PE	
L9	n.i	Pre 1950s ?	✓	✓	✓			Composition, NC	
L11	1487-1, Reliable, Made in Canada	1960s	✓	✓				PE, PVC	
L13	On back of head and second on back of torso, both read: '©The Edward Mobley Co. 1959, MFG. By Arrow Rubber & Plastic Corp."	1959	✓	✓	✓		✓	PVC	

Table S2. Summary of FTIR and PyGCMS analysis of selected samples.

Doll and sample	Sample Description	Identification from FTIR	PyGCMS				
			Retention time (min)	Base Peak	Selected peaks from mass spectrum (m/z)	Compound identified	Identified polymer types and/or additives
A8 S1	Soft flexible plastic of head	PVC Kaolin	3.573	55	28, 39, 41, 84	Cyclopentanone	Adipic acid and bis(2-ethylhexyl) phthalate plasticisers
			14.614	149	39, 50, 65, 76, 93, 105, 121, 135, 177, 195, 222	Diethyl phthalate (DEP)	
			23.476	149	43, 57, 167, 279	Bis(2-ethylhexyl) phthalate	
A8 S2	Migrated plasticiser on head	PVC	3.573	55	41, 84	Cyclopentanone	Adipic acid and low relative amounts of DEP plasticisers
			14.616	149	50, 65, 76, 93, 105, 121, 177	DEP	
C13 S3	Crystals on finger of doll	Phenyl Phosphate	22.329	326	65, 77, 94, 141, 169, 215	Triphenyl phosphate (TPP)	TPP as plasticiser and/or flame retardant
C13 S6	Degraded leg plastic of doll	CA	14.612	149	50, 65, 76, 105, 121, 177, 222	DEP	TPP and DEP as plasticisers and/or flame retardants
			22.329	326	51, 65, 77, 94, 115, 141, 170, 249	TPP	
E6 S1	Flexible plastic of head	PVC Chalk	2.132	41	39, 44, 49, 76, 78	1-Propene, 1-chloro-	Polyvinyl chloride polymer<br>Bis(2-ethylhexyl) phthalate plasticizer<br>Trace of triethylene glycol di(2-ethylhexoate) plasticizer<br>Low relative amount of TPP.
			22.323	326	39, 51, 65, 77, 94, 141, 170, 215, 215, 233, 249	TPP<br>(trace amount)	
			23.524	149	41, 57, 71, 113, 167, 279, (391?)	Bis(2-ethylhexyl) phthalate	
			22.913	171	43, 57, 70, 99, 114, 127, 214, 230,	Triethylene glycol di(2-ethylhexoate) (tentative)	
H1 S1	Hot glue used to adhere feet to the floor		2.048	43	37, 41, 58	Acetone	Ethylene-vinyl acetate copolymer (EVA)[‡].
			2.259	43	43, 45, 60,	Acetic acid	
		Poly(ethylene vinyl acetate)	*A series of C6-C26 hydrocarbon triplet peaks, each consisting of a diene, alkene (major peak) and alkane.*<br>*(see supplementary information)*				
H7 S1			14.326	243	43, 55, 61, 69, 73, 75, 83, 89, 97, 103	1-Dodecanol, TMS derivative	Dominated by stearic acid, lubricant

Doll and sample	Sample Description	Identification from FTIR	PyGCMS				
			Retention time (min)	Base Peak	Selected peaks from mass spectrum (m/z)	Compound identified	Identified polymer types and/or additives
	White crystalline bloom from right hand	Saturated fatty acid (stearic acid?)	15.271	117	43, 55, 69, 73, 75, 257, 272	Dodecanoic acid, TMS derivative	Smaller relative amounts of dodecanoic acid, dodecanol, and palmitic acid.<br>Trace amounts of bis(2-ethylhexyl) phthalate plasticiser
			19.268	73	117, 132, 143, 313, 328	Palmitic acid, TMS derivative	
			21.031	117	43, 55, 73, 145, 201, 341, 356	Stearic acid, TMS derivative (major)	
			23.467	149	41, 57, 70, 83, 113, 167, 279	Bis(2-ethylhexyl) phthalate	
H7, S2	Plastic of right arm	PVC<br>Chalk	6.725	57	41, 43, 55, 99, 119, 126	Heptane, 3-(chloromethyl)-(tentative)	PVC polymer<br>Bis(2-ethylhexyl) phthalate plasticiser
			23.510	149	43, 57, 71, 83, 113, 132, 167, 179, 279, 390	Bis(2-ethylhexyl) phthalate	
J4, S3	Pink paint from back of right leg	Nitrocellulose	22.324	326	39, 51, 65, 77, 94, 170, 215, 233	TPP	TPP as plasticiser and/or flame retardant
K2, S2	Crystals on right arm of CA doll	TPP	6.323	94	39, 66	Phenol	TPP as plasticiser and/or flame retardant
			22.324	326	39, 51, 65, 77, 94, 170, 215, 233	TPP (dominant)	
K2, S3	Flexible plastic of head	PVC	14.610	149	50, 65, 76, 93, 105, 121, 177, 194, 222	DEP	Sample dominated by phthalate plasticisers<br>Bis(2-ethylhexyl) phthalate
			23.465	149	39, 43, 57, 71, 113, 167, 279	Bis(2-ethylhexyl) phthalate	
K3, S1	Plastic of torso	N/A	4.909	104	39, 51, 78	Styrene (dominant)	PS (polymer)<br>Low relative amount of phthalate plasticiser
			11.176	104	50, 74, 76,	Phthalic anhydride	
			14.608	149	65, 76, 105, 121, 132, 177, 222	DEP	
			18.457	149	56, 65, 76, 93, 104, 121, 205, 223	Dibutyl phthalate	
K14, S1	Pink transparent bag accessory with sticky exudate	PVC	23.571	149	41, 43, 57, 71, 83, 93, 104, 113, 132, 167, 279, 390	Bis(2-ethylhexyl) phthalate	Bis(2-ethylhexyl) phthalate plasticiser

Doll and sample	Sample Description	Identification from FTIR	PyGCMS				
			Retention time (min)	Base Peak	Selected peaks from mass spectrum (m/z)	Compound identified	Identified polymer types and/or additives
K14, S2	Colourless transparent bag accessory with no sticky exudate	PVC	14.629	149	39, 50, 65, 76, 93, 105, 121, 132, 177, 194, 222	DEP	DEP and Bis(2-ethylhexyl) phthalate plasticisers
			23.463	149	43, 57, 71, 83, 113, 132, 167, 279	Bis(2-ethylhexyl) phthalate	
			25.247	149	43, 57, 71, 85,97, 127, 167, 293	Phthalate plasticiser (not identified)	
L13, S1	Dark pink plastic from trunks of doll	PVC titanium white	1.954	50	35, 47, 49, 52	Chloromethane	PVC (polymer)<br>DEP plasticiser<br>Phthalic anhydride
			11.155	104	38, 50, 76, 104, 148	Phthalic anhydride	
			14.607	149	50, 65, 76, 93, 105, 121, 177, 132, 149, 177, 222	DEP	

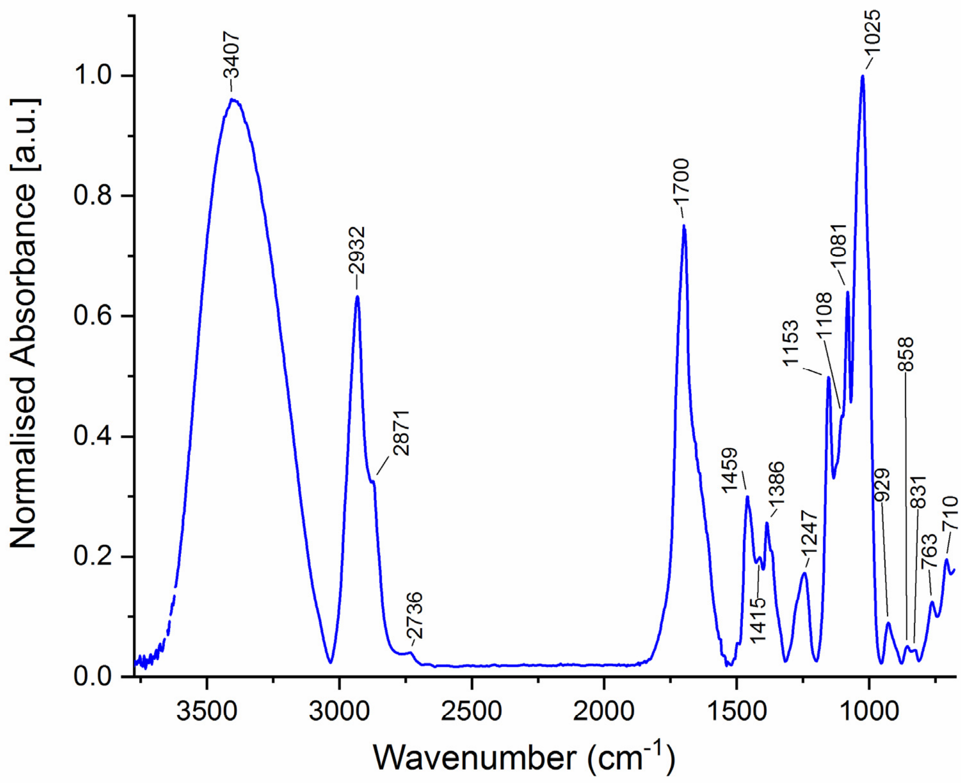

Figure S3. Normalised transmission FTIR analysis of a bulk composition sample from a damage of doll I10. Cellulosic material and a natural resin were identified (see Table S3).

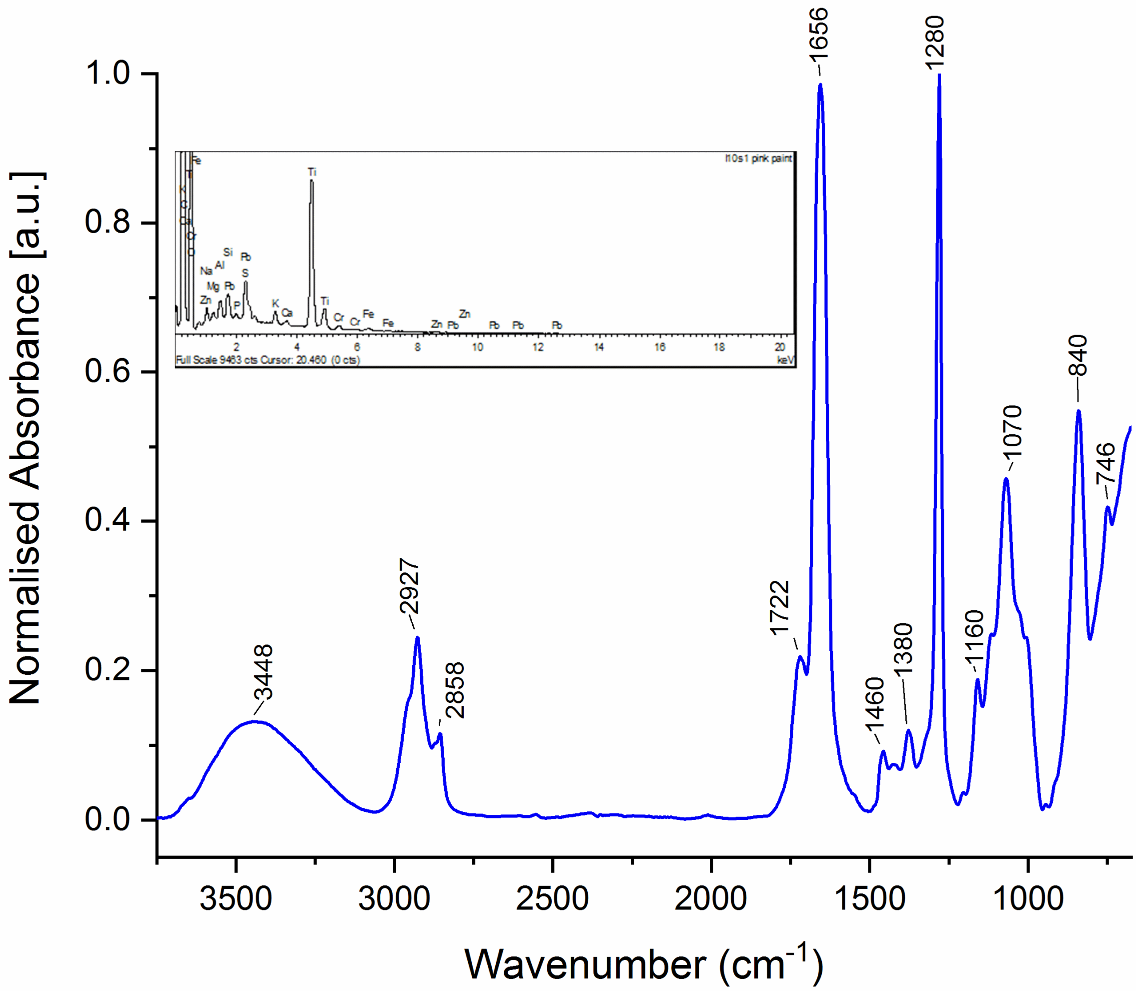

Figure S4. Normalised transmission FTIR and EDX spectrum (shown as insert) of the nitrocellulose pink layer of paint on doll I10. For band assignments see Table S3.

Table S3. Band assignments for FTIR spectra shown in Fig. S3 and Fig. S4

**Spectrum**	**Absorption band (cm^{-1}) assignments**	**Identified component**
Fig. S3 Bulk composition mixture, doll I10	3407 (O-H stretch of OH groups with hydrogen bonding); 2932 (asymmetric stretch CH_2), 2871 & 2736 (symmetric stretch CH_3, CH_2); 1700 (C=O stretch of COOH); 1244 (C-O deformation of bonded OH group); 1459 (C-CH3 asymmetric bend); C-CH_3 (symmetric bend, umbrella mode)	Natural resin
	3407 (O-H stretch of OH groups with hydrogen bonding); 1153 (C-O-C asymmetric vibration); 1108 (Glucose ring stretch, asymmetric; 1025 (C-O stretch), 710 (CH_2 rocking)	Cellulose
Fig. S4 Paint layer, doll I10	1722 (C=O) indicating camphor; 1656 (asymmetric stretch NO_2); 1460 (CH_2 bend); 1280 (NO_2 stretch); 1070 (C-O-C stretch); 840 (N-O stretch); 746 (NO_2 bend)	Nitrocellulose Camphor
	Rise toward the end of the spectrum.	Titanium white

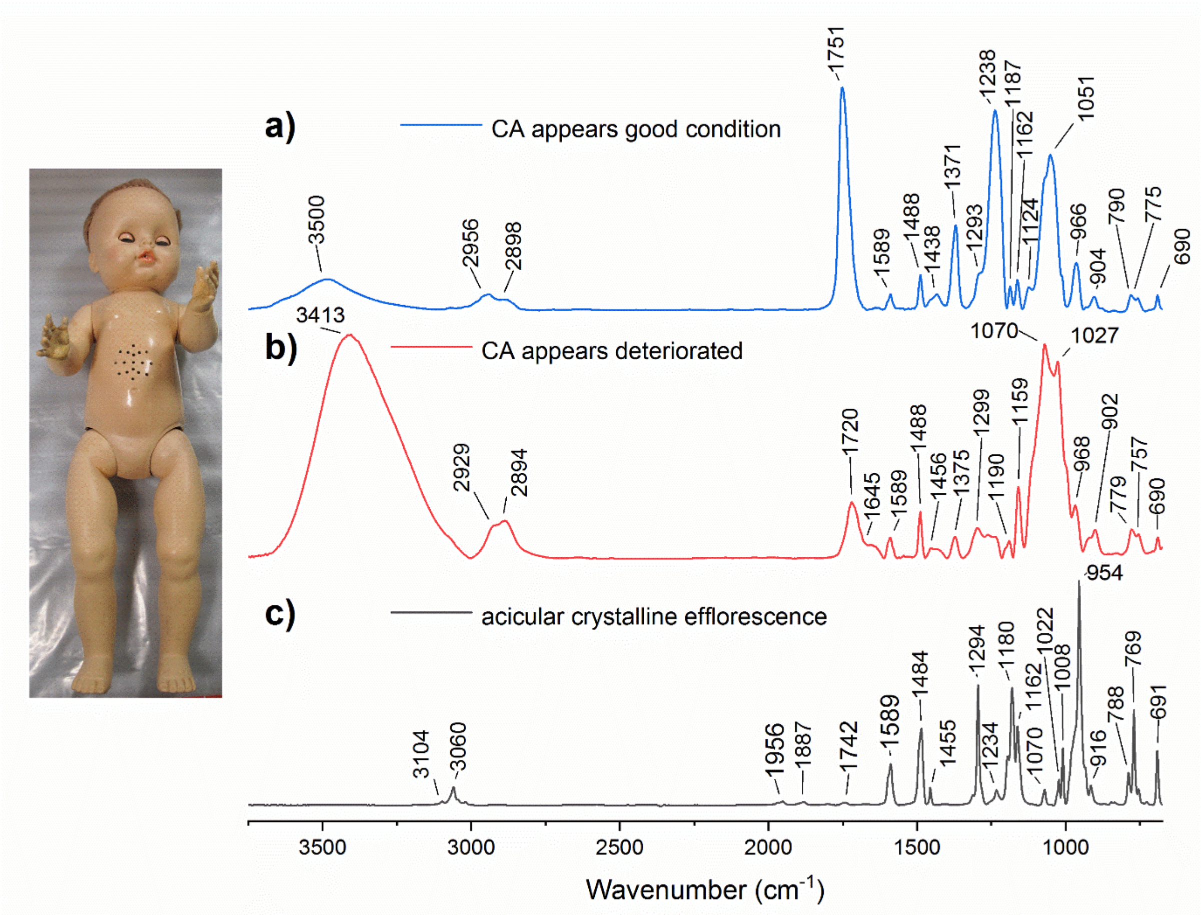

Figure S5. Normalised transmission FTIR spectra of samples taken from cellulose acetate doll C13 (a) cellulose acetate sample from edge of sound hole, which appeared to be in a good condition; (b) cellulose acetate from degraded hip area with powdery and greyed surface; (c) acicular crystalline efflorescence from hand. Image ©Tate.

Table S4. Band assignments for the IR spectra presented in Fig. S5 above

**Absorption band (cm^{-1}) assignments**	**Identified component**
A: 1751 (C=O ester stretch); 1238 (C-C-O ester group, asymmetric bend); 1051 (O-C-C stretch, ester); 1371 (methyl groups in acetate esters). Bands at 1589, 1488, 1293, 1187, 1124, 966 cm^{-1} suggest triphenyl phosphate.	Cellulose acetate<br>Triphenyl phosphate
B: 3413 (OH stretch); relative to spectrum shown in (A), shifted C=O peak from 1751 to 1720; loss of ester bands at 1238 and 1051.	Degraded cellulose acetate
C: 3104, 3060 (aromatic C-H stretch); 1589 (C=C stretch), 1484 (benzene ring modes); 1294 (P=O stretch); 954 (P-O-C), 769 (C-H out-of-plane bend)	Triphenyl phosphate

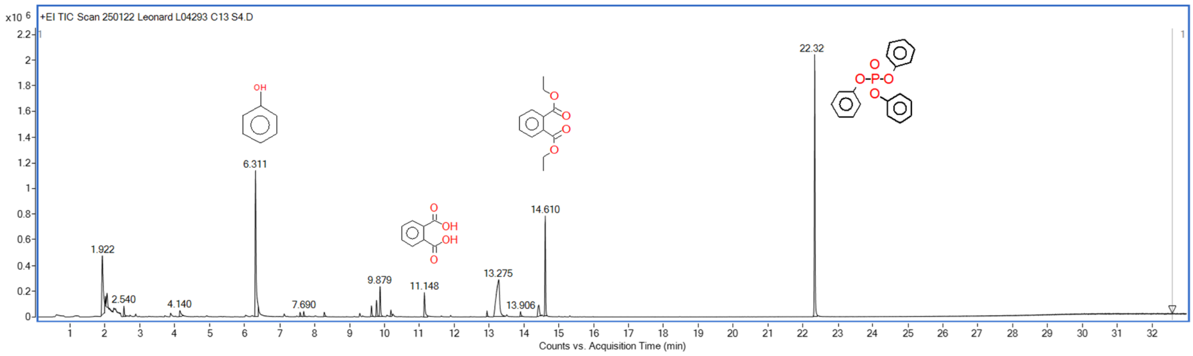

Figure S6. PyGCMS anlaysis of hip plastic from CA doll C13. TPP and DEP plasticisers were identified.

Table S5. Peak assignments for the pyrogram shown in Fig. S6

**Retention time (mins)**	**Base peak**	**Characteristic ions (m/z)**	**Identified compound**
1.920	44	--	Carbon dioxide
2.540	78	39, 51	Benzene
4.140	95, 96	39	Furfural
6.311	94	39, 66	Phenol
7.587	81	39, 53, 97, 105	3-Cyclopentene-1,2-diol, cis- (tentative)
7.690	68	39, 57, 81, 97, 105	Unidentified
8.275	68	39, 42, 53, 68, 81, 96, 98, 123	Levoglucosenone
9.632	69	57, 85, 86, 98, 114, 144	1,4:3,6-Dianhydro-α-d-glucopyranose
9.776	43	57, 71, 85, 97, 144	3,4-Anhydro-d-galactosan
9.879	71	41, 43, 69, 81, 97, 115	2,3-Anhydro-d-mannosan (Tentative)
11.148	104	50, 76, 148	1,2-Benzenedicarboxylic acid
12.942	163	50, 77, 92, 135, 149, 194	Dimethyl phthalate
13.275	60	57, 73, 98, 115, 126, 144	β-D-Glucopyranose, 1,6-anhydro-
13.906	43	60, 69, 85, 98, 115, 143	Unidentified
14.427	73	43, 69, 85, 98, 115, 131, 192	1,6-Anhydro-β-D-glucofuranose (tentative)
14.610	149	50, 65, 76, 93, 105, 121, 177, 194,	Diethyl phthalate (DEP)
22.329	326	39, 51, 65, 77, 94, 115, 141, 169,	Triphenyl phosphate (TPP)

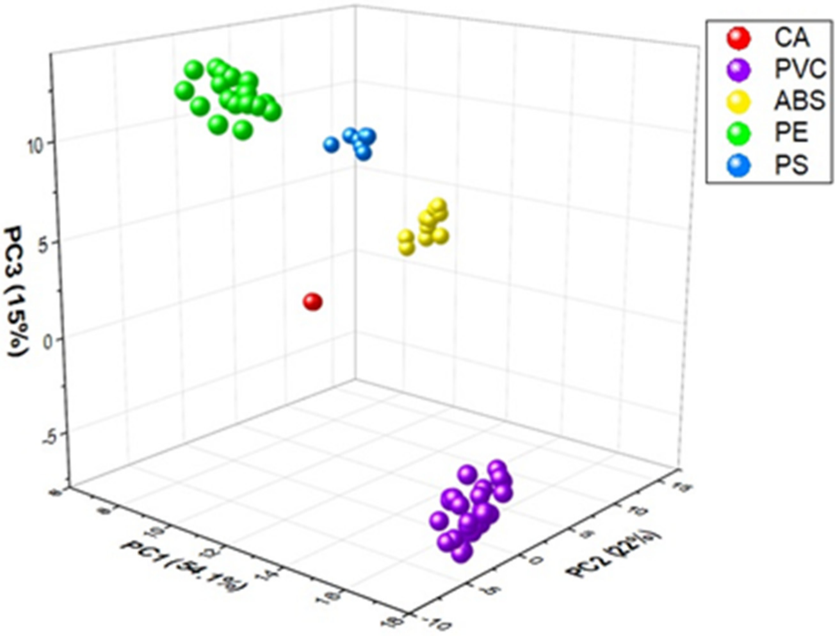

Figure S7. 3D Scatter plot of PCA analysis of various dolls analysed using NIR.

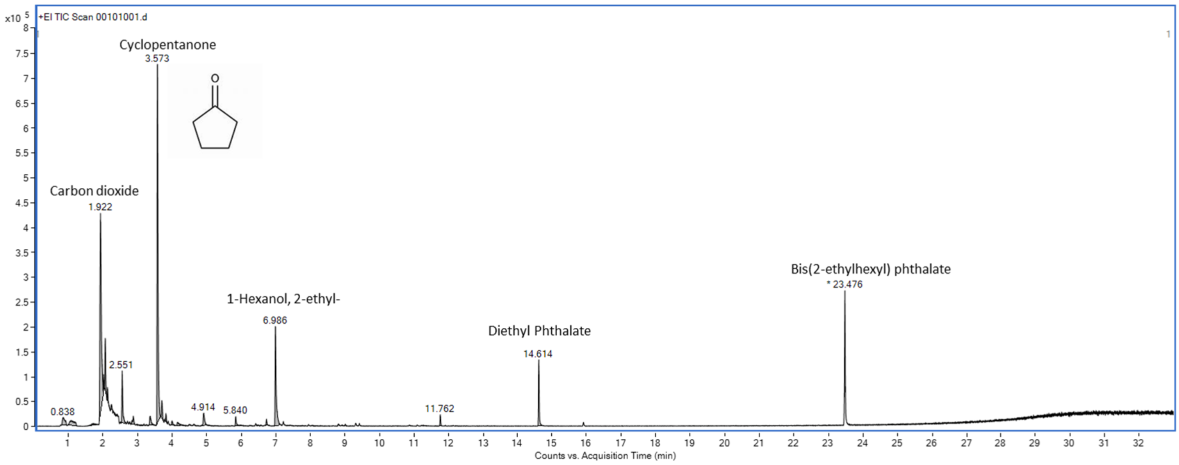

Figure S8. PyGCMS analysis of a sample of the flexible plastic used to construct the head of doll A8. Dominant pyrolysates were cyclopentanone, and di(2-ethylhexyl)phthalate.

Table S6. Peak assignments for the pyrogram shown in Fig. S8.

Retention time (mins)	Base peak	Characteristic ions (m/z)	Identified Compound
1.922	44	27, 42	Carbon dioxide
2.011	58	38, 42, 58	Not identified
2.060	58	39, 43, 58	Propylene oxide (?) M
2.126	41	39, 41, 49, 76, 78	1-Propene, 2-chloro
2.551	78	39, 50	Benzene
2.872	69	39, 41, 100	Methyl methacrylate
3.355	91	92, 51, 55, 77	Toluene
3.573	55	28, 39, 41, 84	Cyclopentanone
3.697	55	27, 41, 69, 83, 112	3-Ethyl-2-hexene (?)
3.816	70	27, 41, 55, 70, 82, 112	2-Heptene, 3-methyl-
4.914	104	51, 63, 78	Styrene
5.840	57	14, 29, 43, 72, 128	Hexanal, 2-ethyl-
6.986	57	41, 70, 83, 98, 112	Not identified
9.303	128	102	Azulene
11.760	142	41, 55, 84, 112	Not identified
14.614	149	39, 50, 65, 76, 93, 105,	Diethyl Phthalate
15.907	105	70, 77, 112	Benzoic acid, 2-ethylhexyl
23.476	149	43, 57, 167, 279	Bis(2-ethylhexyl)

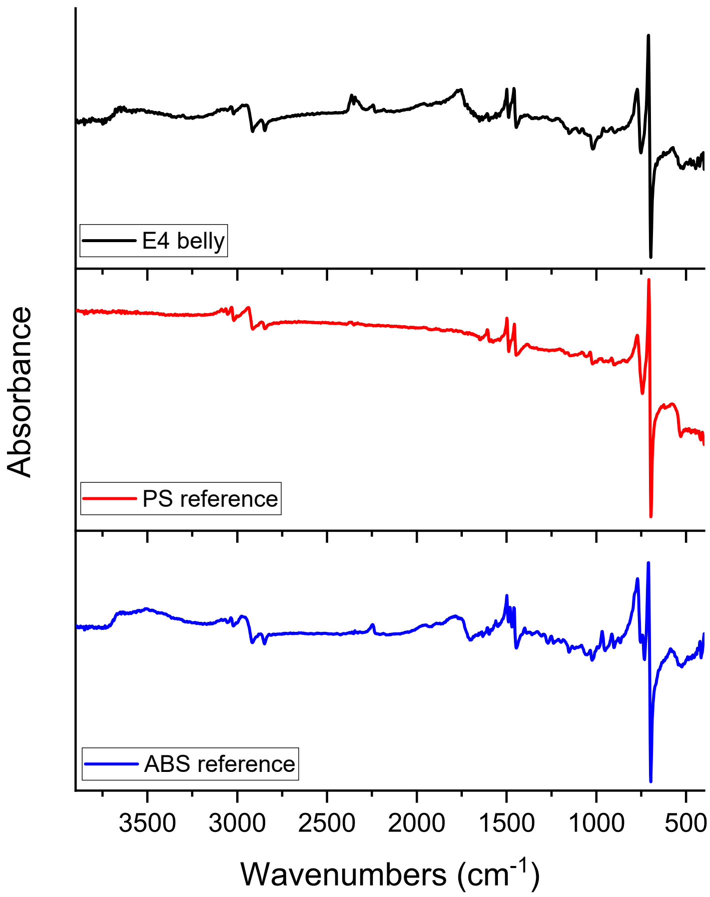

Figure S9. Comparison of ER-FTIR spectra of doll E4, to ER-FTIR reference spectra for PS and ABS. The ER-FTIR spectra of PS and ABS appear closely similar, however a nitrile band at ~2250 cm^{-1} helps distinguish between the two polymers.

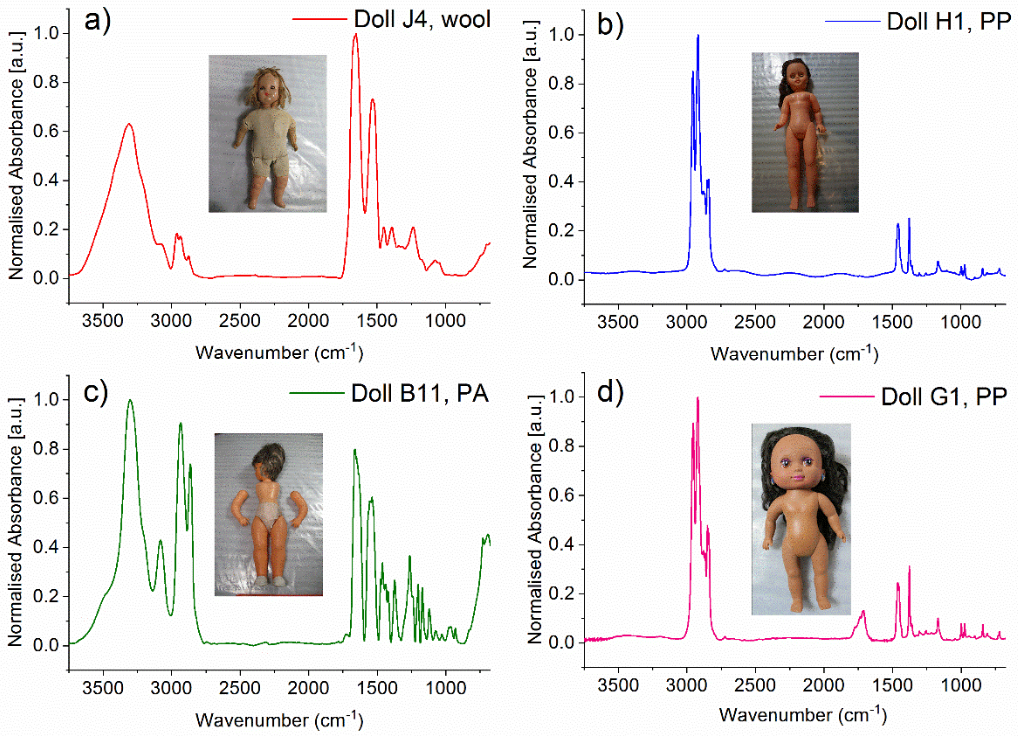

Figure S10. Normalised transmission FTIR spectra of hair fibres from four dolls: **(a)** matted woollen hair fibres of doll J4; **(b)** Friable and fragmenting PP hair fibres from doll H1; **(c)** Friable and fragmenting polyamide fibres from doll B11; **(d)** Friable and fragmenting PP hair fibres from doll G1. Images ©Tate.

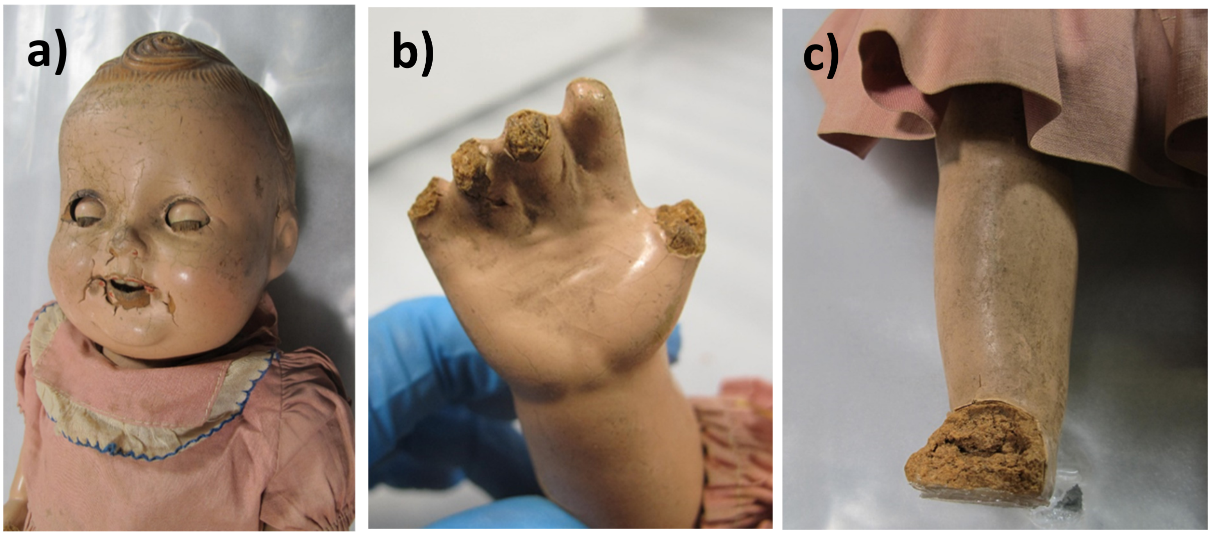

Figure S11. Details of composition doll D11 showing cracking and lifting paint surface, and multiple losses **(a)** head with lifting paint **(b)** missing finger tips showing the underlying brown material that forms the shape of the doll. **(c)** similar to **(b)** but partial loss of foot. Image ©Tate.

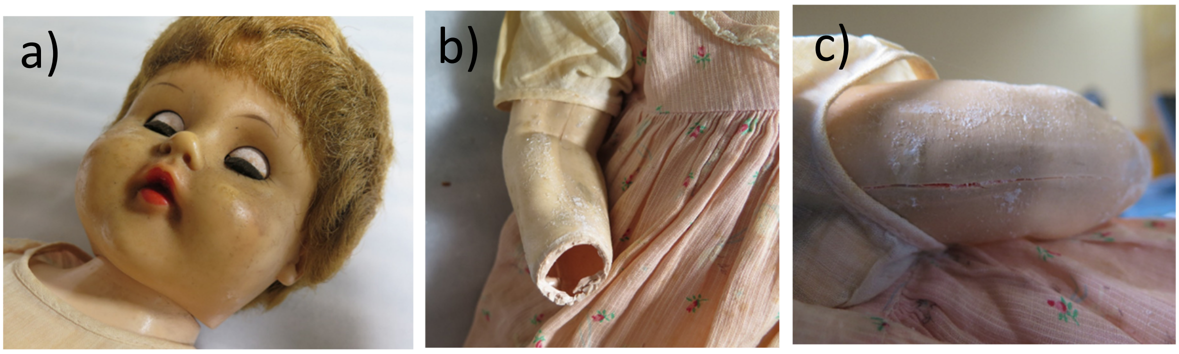

Figure S12. Details of doll K2 showing **(a)** sweating of the PVC head **(b)** cracking and loss to the CA arm **(c)** crystal deposits on surface of arm. Image ©Tate.

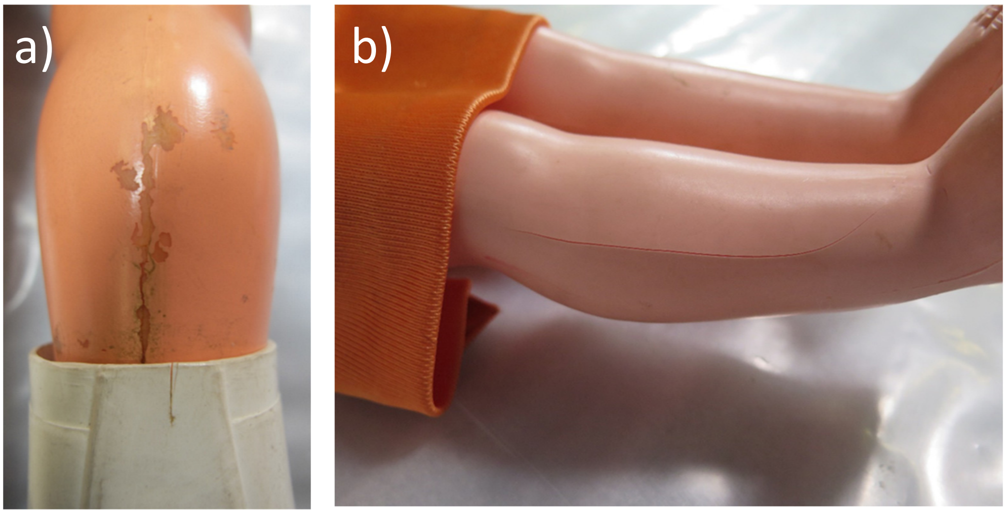

Figure S13. **(a)** Details of dolls G11 PS leg with flaking NC paint layer **(b)** Doll I14, showing cracking of (likely PE) legs which may worsen during handling and display. Image ©Tate.

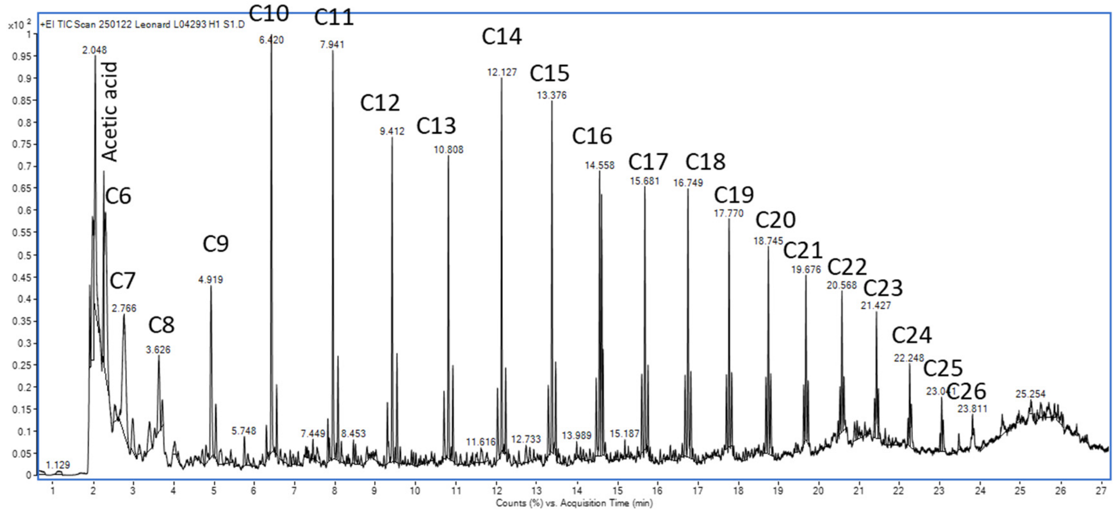

Figure S14. PyGCMS of a sample of hot glue taken from underneath the foot of Doll H1. A series of C6-C26 hydrocarbon triplet peaks, each consisting of a diene, alkene (major peak) and alkane. Acetic acid was also identified at 2.259 min. This is consistent with ethylene-vinyl acetate copolymer (EVA).
